# Synthesis and Anticancer Activities of Novel Guanylhydrazone and Aminoguanidine Tetrahydropyran Derivatives

**DOI:** 10.3390/molecules21060671

**Published:** 2016-06-21

**Authors:** Fábio Pedrosa Lins Silva, Bruna Braga Dantas, Gláucia Veríssimo Faheina Martins, Demétrius Antônio Machado de Araújo, Mário Luiz Araújo de Almeida Vasconcellos

**Affiliations:** 1 Departamento de Química, Campus I, Laboratório de Síntese Orgânica Medicinal da Paraíba (LASOM-PB), Universidade Federal da Paraíba, João Pessoa, CEP:58051-900, Paraíba, Brazil; pedrosalinssilva@gmail.com; 2 Departamento de Biotecnologia, Campus I, Laboratório de Biotecnologia Celular e Molecular, Universidade Federal da Paraíba, João Pessoa, CEP:58051-900, Paraíba, Brazil; brunabdantas@gmail.com (B.B.D.); glauciafaheina@yahoo.com.br (G.V.F.M.); demetrius@cbiotec.ufpb.br (D.A.M.A.)

**Keywords:** Prins cyclization reaction, tetraydropyran derivatives, anticancer, guanylhydrazone, aminoguanidine

## Abstract

In this paper we present the convenient syntheses of six new guanylhydrazone and aminoguanidine tetrahydropyran derivatives **2**–**7**. The guanylhydrazone **2**, **3** and **4** were prepared in 100% yield, starting from corresponding aromatic ketones **8a**–**c** and aminoguanidine hydrochloride accessed by microwave irradiation. The aminoguanidine **5**, **6** and **7** were prepared by reduction of guanylhydrazone **2**–**4** with sodium cyanoborohydride (94% yield of **5**, and 100% yield of **6** and **7**). The aromatic ketones **8a**–**c** were prepared from the Barbier reaction followed by the Prins cyclization reaction (two steps, 63%–65% and 95%–98%). Cytotoxicity studies have demonstrated the effects of compounds **2**–**7** in various cancer and normal cell lines. That way, we showed that these compounds decreased cell viabilities in a micromolar range, and from all the compounds tested we can state that, at least, compound **3** can be considered a promising molecule for target-directed drug design.

## 1. Introduction

Despite the availability of improved drugs, including targeted cancer therapies, cancer is still one of the leading causes of mortality worldwide. It is estimated to have accounted for 8.2 million deaths (around 13% of all deaths) in 2007, and ~1.4 million new cancer cases and ~566,000 deaths from cancer occurred in the United States in 2008 [1,2,3,4]. Additionally, a 70% increase in new cases of cancer is expected over the next two decades. Therefore, new, more effective treatment strategies are required to address this issue. A substantial number of new antineoplastic agents have been discovered. Considerable insight has been gained into the mechanisms by which many of these compounds affect cellular growth and this knowledge has been used in the design of new chemotherapeutic drugs [5].

**Figure 1:**
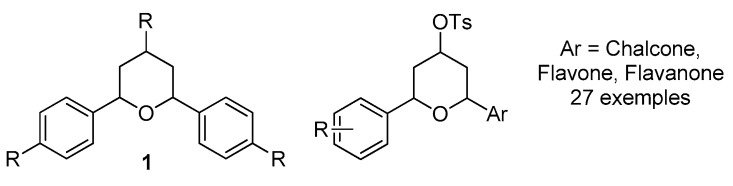
The compound 2,4,6-Diaryltetraydropyran has antiproliferative activity against human cancer cell lines.

In recent years, several compounds based on the tetrahydropyran moiety have been described as presenting high anticancer activities [6,7,8,9,10,11]. Substituted tetrahydropyranyl moieties are extensively distributed in the natural products’ structures that present a large pharmacological profile [12]. There are many synthetic methodologies to prepare this moiety, *i.e.*, the Prins cyclization reaction and others. In 2014, Ahmed *et al.* described several 2,4,6-diaryltetraydropyran scaffolds that possess immense importance with antiproliferative activity against human cancer cell lines (Figure 1) [13].

**Figure 2:**
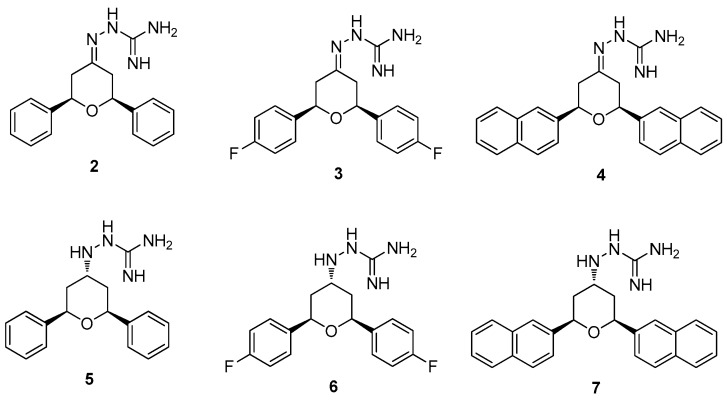
Compounds synthesized and evaluated against tumor cells line in this work.

In connection to our interest in the stereoselective synthesis of tetraydropyran derivatives using the Prins cyclization reaction [14,15,16,17,18], and considering that guanylhydrazone [19] and aminoguanidine groups [20,21] potentiate the anticancer activity of some drugs, and these nitrogenated bases could be considered pharmacophoric points, we present in this article the syntheses of six novel tetrahydropyranyl guanylhydrazone and aminoguanidine **2**–**7** (Figure 2). That way, we performed *in vitro* evaluation for anticancer activity against cancer cell lines, such as the chronic myeloid leukemia (K562), human acute myeloid leukemia (HL-60), human breast adenocarcinoma (MCF-7), human colon adenocarcinoma (HT-29), and L929 (murine fibroblast) and the human peripheral blood of patients with chronic myeloid leukemia (PBMC/CML) cells. Normal cell lines, L929 (murine fibroblast) cells and human peripheral blood cells (PBMC) were also evaluated.

It is important to note that compounds **2**–**7** are Meso compounds which greatly simplifies these syntheses, since only relative configuration (*cis*\*trans*) must be controlled [12].

## 2. Results and Discussion

### 2.1. Chemistry

**Scheme 1:**
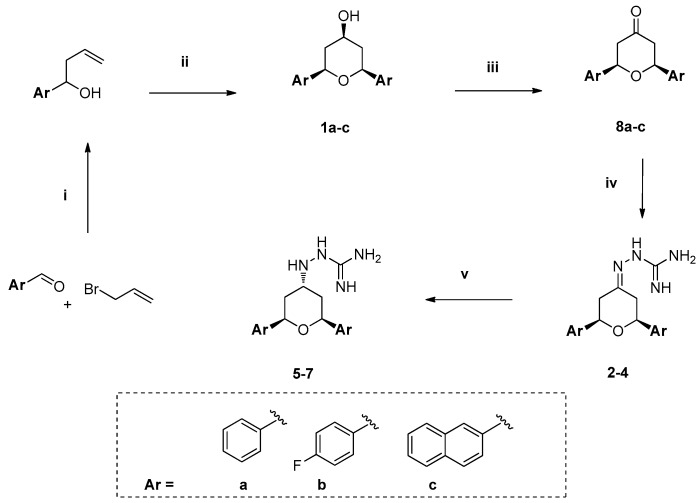
Synthesis of guanylhydrazone **2**–**4** and aminoguanidine **5**–**7**. (i) SnCl_2_, KI, H_2_O, NH_4_Cl, 0 °C, 2 h, r.t., 96%–98% yields; (ii) 1-PhH, AcOH, BF_3_·Et_2_O, 0 °C, 3 h, r.t.; 2-K_2_CO_3_, MeOH, 30 min, r.t., 60%–63% yields; (iii) PCC, CH_2_Cl_2_, r.t., 2.5 h , 95%–98% yields; (iv) aminoguanidine hydrochloride, EtOH, microwave, 100 °C, 5 min, 100% yields; (v) NaCNBH_3_, 0°C, 2 h, r.t., 94%–100% yields.

Our experimental work starts with the preparation in good yield of homoallylic alcohols shown in Scheme 1 (step i) from the Barbier reaction [22] between the corresponding aldehydes (benzaldehyde, 4-fluorobenzaldehyde and 2-naphthaldehyde) with allyl bromide in the presence of stannous chloride (96%–98% yields). In step ii the corresponding tetrahydropyran rings of **1a**–**c** are prepared with control of relative configuration (2,4,6-cis) from the Prins cyclization reaction [12], followed by hydrolysis of the acetyl intermediate with potassium carbonate in methanol (60%–63% yields). The cis stereoselectivity of these reactions is anticipated based on the proposed mechanisms 4 for these reactions and determined by bi-dimensional spectroscopic study (NOESY spectra, see in Supplementary Materials) and by X-ray crystallography of **1** [23]. The oxidation of tetrahydropyran alcohols to the corresponding ketones **8a**–**c** (step iii, Scheme 1) was performed in high yields by using PCC in dichloromethane as a solvent (95%–98% yields). The guanylhydrazone **2**–**4** (Figure 1) were prepared in quantitative yields (100% yields) by reacting ketones **8a**–**c** with aminoguanidine hydrochloride and ethanol as a solvent (no catalysts were used) promoted by 5 min of microwave irradiation (250 W) at 100 °C (step iv). Finally, the reductions of aminoguanidine **2**–**4** were performed in very high yields (94%–100% yields) by reacting **2**–**4** with sodium cyanoborohydride in ethanol as a solvent at room temperature (step v, Scheme 1). The relative configurations of C4 of aminoguanidine **5**–**7** were determined by ^1^H-NMR spectroscopic studies (NOESY spectrum, see in Supplementary Materials).

### 2.2. Biology

The anticancer drug discovery screening has been designed to distinguish between broad-spectrum anticancer compounds and cancer-selective agents [24]. The anticancer activities of **2**–**7** were investigated against different types of cancer cells and non-cancer cells using MTT assay. Each cell line was incubated with different concentrations (3–100 µmol·L^−1^) for each compound and this was used to create a concentration-response relationship curve. The response parameter (IC_50_) was calculated for each cell line. The IC_50_ value corresponds to the compound’s concentration causing 50% of reduction viability at the end of the incubation period (24 or 72 h). Compounds **2**–**7** indicated potency anticancer activity against K562, HL-60, MCF-7, HT-29 and PBMC/CML cell lines. The analogues showed a broad-spectrum anticancer activity independent of the incubation period (Table 1 and Table 2).

The effects of compounds **3** and **7**, which were the most promissory substances, had similar action on K562 and PBMC/CML cells. Moreover, those cells showed to be the most sensitive cells. It is important because those types of chronic myeloid leukemia have expressed the BCR-ABL gene, which stimulates cell growth, and it is highly resistant to anticancer therapy [25,26]. Therefore, our result can address an association with a possible clinical application.

In this work, etoposide has been used as the reference substance, because this compound is known is a standard drug used in the treatment of a wide spectrum of solid tumors, lymphomas and leukemias. It is a topoactive drug which inhibits topoisomerase II–DNA cleavable complex, resulting in DNA damage, and often results in cell death apoptosis [27,28].

The results have demonstrated that DNA is vulnerable to damage after treatment with compounds **3** and **7**, similar to therapeutics such as etoposide. Many anticancer drugs exert their effect on the cell cycle which is known as being cell-cycle-specific. The compounds **3** and **7** induced cell-cycle arrest (Supplement Figures S1 and S2) in the G1 phase, corresponding to IC_50_ in 24 h, as well as an increase of cells in the sub-G1 phase, representing sub diploid content that corresponds to apoptotic cells with fragmented DNA or condensed chromatin [29]. This event demonstrates that the addition of the compound inhibits the cell cycle and induces cell death by apoptosis, probably because these cells were unable to repair the damage caused by molecules [30].

Our results also showed that the different substituents added around the tetrahydropyran ring were critical to the motifs’ efficacy and our cytotoxic data showed that compounds **2**, **3** and **4** are, at least, two-fold more potent than compounds **5**, **6** and **7** (Table 1 and Table 2). The analogs **3** and **6** become more powerful than **2** and **5**, probably due to the addition of the fluor atom at position **4** in the aromatic ring. Compounds **4** and **7** were even more potent than the other analogs, probably due to the presence of two naphthyl rings, which alter the molecular properties, *i.e.*, lipophilicity, among others. Other studies corroborate with our results, demonstrating increased activity of tetrahydropyran derivates with a further lipophilicity group [31].

The six compounds **2**–**7** demonstrated potent anticancer activity. Moreover, compound **3** showed the most promising effects due to both its powerful cytotoxic activity in K562 cells and being the most selective compound tested [32].

## 3. Materials and Methods

### 3.1. Chemistry

#### 3.1.1. General Methods

All commercially available reagents were purchased from Aldrich and used without further purification. Reactions were monitored by thin-layer chromatography (TLC) using Silica gel 60 UV254 Macherey-Nagel pre-coated silica gel plates; detection was by means of a UV lamp and revelation to vanillin. Flash column chromatography was performed on 300–400 mesh silica gel. Organic layers were dried over anhydrous MgSO_4_ or Na_2_SO_4_ prior to evaporation on a rotary evaporator. The reactions performed under microwave irradiation were performed in a microwave reactor model CEM Discover System-Benchmate (CEM Corporation, 3100 Smith Farm Road Matthews, NC. USA, 28104) quipped with a system for continuous irradiation with programmable μW power in the range from 0 to 300 W, with temperature monitored by infrared sensor. ^1^H- and ^13^C-NMR spectra were obtained by using a Varian Mercury Spectra AC 200 (200 MHz for ^1^H and 50 M for ^13^C) (Varian, Palo Alto, CA, USA) or in CDCl_3_, DMSO or CD_3_OD. Spectral patterns are designated as s singlet; d doublet; dd doublet of doublets; ddd double doublet of doublets; t triplet; dt double triplet; q quartet; m multiplet. The chemical shifts (δ, ppm) were reported in values relative to tetramethylsilane (0 ppm) for ^1^H-NMR and were reported in the scale relative to CDCl_3_ (77.00 ppm), DMSO (39.52 ppm) or CD_3_OD (49.00 ppm) for ^13^C-NMR and coupling constants (*J*) were expressed in. The Fourier Transform Infrared Spectroscopy spectra were obtained using a spectrophotometer IR-Prestige-21 (Shimadzu, Kyoto, Japan). MS data were measured with a Shimadzu GCMS–QP2010 mass spectrometer (Shimadzu). The elementary analyses of unpublished compounds were performed in an analyzer organic elemental CHNS-O, FLASH 2000 of Perkin-Elmer, Waltham, MA, USA).

#### 3.1.2. General Procedure for Homoallylic Alcohol Synthesis

The synthesis of homoallylic alcohol was performed through a Barbier reaction. The corresponding aldehyde (8 mmol) was added to water (40 mL) containing potassium iodide (24 mmol), stannous chloride dehydrate (12 mmol) and allyl bromide (1 mL). The orange solution turns white by addition of saturated ammonium chloride (20 mL). The stirring was continued for 2 h at room temperature. After the end of reaction, the reaction mixture was extracted with CH_2_Cl_2_ (2 × 50 mL) gave an organic phase, washed with water, dried with sodium sulfate anhydrous, concentrated under reduced pressure and purified by flash chromatography on silica gel (EtOAc:Hexane as eluent). The products were concentrated under reduced pressure yielding the homoallylic alcohol, respectively [23].

*1-Phenylbut-3-en-1-ol.* This product was obtained using benzaldehyde. The product was purified by silica gel column chromatography using EtOAc/hexane (1:9) as eluent, resulting in 97% yield. IR (KBr) ν/cm^−1^ 3371, 3070, 3028, 2904, 1639, 1492, 1049, 995, 914, 756, 702; ^1^H-NMR (200 MHz, CD_3_OD) δ 7.33 (m, 5H), 5.83 (m, 1H), 5.18 (m, 2H), 4.74 (t, 1H, *J* = 6.0 Hz), 2.53 (m, 3H); ^13^C-NMR (50 MHz, CDCl_3_) δ 144.57, 135.18, 129.09, 128.22, 126.54, 119.02, 74.03, 44.49.

*1-(4-Fluorophenyl)but-3-en-1-ol.* This product was obtained using 4-fluorobenzaldehyde. The product was purified by silica gel column chromatography using EtOAc/hexane (3:7) as eluent, resulting in 96% yield. IR (KBr) ν/cm^−1^ 3383, 3078, 2981, 2908, 1604, 11508, 1049, 995, 914, 837; ^1^H-NMR (200 MHz, CDCl_3_) δ 7.41 (m, 2H), 7.14 (m, 2H), 5.88 (m, 1H), 5.25 (m, 2H), 4.80 (t, 1H, *J* = 6.0 Hz), 2.57 (m, 3H); ^13^C-NMR (50 MHz, CDCl_3_) δ 165.29, 160.42, 140.31, 140.25, 134.96, 134.88, 128.29, 128.13, 119.36, 116.13, 115.71, 73.45, 73.33, 44.62.

*1-(Naphthalen-2-yl)but-3-en-1-ol.* This product was obtained using 2-naphthaldehyde. The product was purified by silica gel column chromatography using EtOAc/hexane (1:9) as eluent, resulting in 98% yield. IR (KBr) ν /cm^−1^ 3278, 3051, 2947, 2854, 1600, 1508, 1060, 991, 952, 821, 748; ^1^H-NMR (200 MHz, CDCl_3_) δ 7.67 (m, 7H), 5.84 (m, 1H), 5.18 (m, 2H), 4.90 (t, 1H, *J* = 6.0 Hz), 2.60 (m, 2H), 2.34 (s, 1H); ^13^C-NMR (50 MHz, CDCl_3_) δ 141.98, 135.11, 133.98, 133.67, 128.94, 128.70, 128.42, 126.86, 126.55, 125.26, 124.75, 119.23, 74.19, 44.45.

#### 3.1.3. General Procedure for Synthesis of 4-Hydroxy-2,6-diaryl-tetrahydropyran

A round bottom flask equipped with a magnetic stir bar was charged with 0.81 mL of acetic acid, 7 mL of benzene, 4.5 mmol of homoallylic alcohol and 9 mmol of aldehyde. The mixture was cooled to 0 °C and after slow addition of 1.2 mL of boron trifluoride etherate was stirred in this temperature for 3 h. To the reaction media was added saturated NaHCO_3_ in water (10 mL) followed extraction with EtOAc (3 × 10 mL). The combined organic phase are washed with brine (3 × 10 mL), dried over Na_2_SO_4_, filtered and concentrated under vacuum. The acetylated thus obtained in this reaction was dissolved in methanol (10 mL) and stirred over potassium carbonate (500 mg) for 0.5 h. Then methanol was removed under reduced pressure and water (20 mL) was added. The mixture was extracted with EtOAc (2 × 20 mL) and the combined organic layers were dried over anhydrous Na_2_SO_4_ and the solvent was removed under reduced pressure. The resulting crude product was purified by column chromatography on silica gel to give compound 4-Hydroxy-2,6-disubstituted tetrahydropyran [23].

*2,6-Diphenyl-tetrahydro-2H-pyran-4-ol* (**1a**). This product was obtained using 4.5 mmol of 1-phenylbut-3-en-1-ol and 9 mmol of benzaldehyde. The product was purified by silica gel column chromatography using EtOAc/hexane (1:3) as eluent, resulting in 60% yield. IR (KBr) ν/cm^−1^ 3263, 3032, 2947, 2858, 1604, 1496, 1319, 1060, 752, 694; ^1^H-NMR (200 MHz, CDCl_3_) δ 7.37 (m, 10H), 4.57 (d, 2H, *J* = 12.0 Hz), 4.10 (m, 1H), 2.27 (dd, 2H, *J* = 12.0, 4.0 Hz), 2.12 (m, 1H), 1.60 (q, 2H, *J* = 12.0 Hz); ^13^C-NMR (50 MHz, CDCl_3_) δ 142.49, 128.85, 128.02, 126.39, 78.32, 69.03, 43.51.

*2,6-Bis(4-fluorophenyl)-tetrahydro-2H-pyran-4-ol* (**1b**). This product was obtained using 4.5 mmol of 1-(4-fluorophenyl) but-3-en-1-ol and 9 mmol of 4-Fluorobenzaldehyde. The product was purified by silica gel column chromatography using EtOAc/hexane (3:7) as eluent, resulting in 62% yield. IR (KBr) ν/cm^−1^ 3329, 3051, 2943, 2854, 1604, 1512, 1230, 1068, 821; ^1^H-NMR (200 MHz, CDCl_3_) δ 7.33 (m, 4H), 7.04 (m, 4H), 4.53 (d, 2H, *J* = 12.0 Hz), 4.09 (m, 1H), 2.23 (m, 2H), 2.02 (s, 1H), 1.56 (dd, 2H, *J* = 14.0, 12.0 Hz); ^13^C-NMR (50 MHz, CDCl_3_) δ 165.36, 160.48, 138.39, 138.33, 128.41, 128.25, 116.18, 115.75, 78.14, 78.06, 69.09, 43.70.

*2,6-Di(naphthalen-2-yl)-tetrahydro-2H-pyran-4-ol* (**1c**). This product was obtained using 4.5mmol of 1-(naphthalen-2-yl)but-3-en-1-ol and 9 mmol of 2-naphthaldehyde. The product was purified by silica gel column chromatography using EtOAc/hexane (1:1) as eluent, resulting in 63% yield. IR (KBr) ν/cm^−1^ 3278, 3020, 2947, 2854, 1600, 1508, 1357, 1060, 821, 748; ^1^H-NMR (500 MHz, DMSO) δ 7.92 (m, 8H), 7.63 (dd, 2H, *J* = 10.0, 5.0 Hz), 7.50 (m, 4H), 4.82 (d, 2H, *J* = 10.0 Hz), 4.11 (m, 1H), 3.82 (s, 1H), 2.29 (dd, 2H, *J* = 10.0, 5.0 Hz), 1.58 (dd, 2H, *J* = 25.0, 10.0 Hz); ^13^C-NMR (125 MHz, DMSO) δ 140.76, 133.40, 132.94, 128.41, 128.30, 128.02, 126.59, 126.28, 125.08, 124.64, 77.81, 67.35, 43.63.

#### 3.1.4. General Procedure for Oxidation of 4-Hydroxy-2,6-diaryl Tetrahydropyrans 

In a typical reaction, 1 mmol of 4-hydroxy-2,6-disubstituted tetrahydropyran was dissolved in 6 mL of CH_2_Cl_2_ dry in one round bottom flask and treated with 1 mmol of PCC. The stirring was continued for 2.5 h at room temperature where the completion of the reaction was monitored by thin-layer chromatography (TLC). The mixture was extracted with CH_2_Cl_2_ and filtered through a short pad of silica gel with hexane and the solvent evaporated to 2,6-disubstituted–tetrahydropyran -4-one. No side products were observed to be formed [23].

*2,6-Diphenyl-tetrahydropyran-4-one* (**8a**). This product was obtained using 1 mmol of 2,6-diphenyl-tetrahydro-2*H*-pyran-4-ol, resulting in 98% yield. IR (KBr) ν/cm^−1^ 3032, 2970, 2858, 1720, 1604, 1496, 1056, 1060, 752, 698; ^1^H-NMR (500 MHz, CDCl_3_) δ 7.40 (m, 10H), 4.85 (dd, 2H, *J* = 10.0, 5.0 Hz), 2.73 (m, 4H); ^13^C-NMR (125 MHz, CDCl_3_) δ 206.39, 141.10, 128.99, 128.43, 126.01, 79.31, 50.06.

*2,6-Bis(4-fluorophenyl)-tetrahydropyran-4-one* (**8b**). This product was obtained using 1 mmol of 2,6-bis(4-fluorophenyl)-tetrahydro-2H-pyran-4-ol, resulting in 97% yield. IR (KBr) ν/cm^-1^ 3070, 2970, 2893,1720, 1608, 1512, 1053, 1138,829; ^1^H-NMR (200 MHz, CDCl_3_) δ 7.41 (m, 4H), 7.08 (m, 4H), 4.82 (dd, 2H, *J* = 10.0, 4.0 Hz), 2.66 (m, 4H); ^13^C-NMR (50 MHz, CDCl_3_) δ 206.25, 165.65, 160.74, 137.14, 137.08, 128.28, 128.12, 116.54, 116.11, 79.12, 50.35.

*2,6-Di(Naphthalen-2-yl)-tetrahydropyran-4-one* (**8c**). Product **8c** was obtained using 1 mmol of 2,6-di(naphthalen-2-yl)-tetrahydro-2*H*-pyran-4-ol, resulting in 95% yield. IR (KBr) ν/cm^−1^ 3051, 2978, 2897, 1708, 1600, 1508, 1045, 825, 748; ^1^H-NMR (200 MHz, CDCl_3_) δ 7.69 (m, 14H), 5.06 (m, 2H), 2.84 (m, 4H); ^13^C-NMR (50 MHz, CDCl_3_) δ 206.76, 138.76, 133.95, 133.85, 129.33, 128.83, 128.48, 127.10, 126.95, 125.38, 124.41, 79.97, 50.42. 

#### 3.1.5. General Procedure for the Preparation of Guanylhydrazone (**2**–**4**)

The hydrazones were prepared by reaction of ketones with aminoguanidine hydrochloride with the aid of a microwave device. A mixture of 2,6-di-substituted-tetrahydropyran-4-one (0.5 mmol) and aminoguanidine hydrochloride (0.5 mmol) in 1 mL of ethanol were placed in a glass tube for specific microwave reactor along with a magnetic. A shaker reaction was performed under microwave irradiation to 100 °C (read monitored by infrared sensor) for 5 min (“Hold Time”) under conditions of a closed vessel. After completion of the reaction, the solvent was evaporated under reduced pressure. This crude product was then subjected to flash column chromatography to yield a solid.

*2-(2,6-Diphenyl-2H-pyran-4(3H)-ylidene)hydrazinecarboximidamide* (**2**). This product was obtained using 0.5 mmol of 2,6-diphenyl-tetrahydropyran-4-one (**8a**). The product was purified by column chromatography on silica gel using methanol/EtOAc (1:9) as eluent, resulting in quantitative yield (100%). IR (KBr) ν/cm^−1^ 3348, 3309, 3155, 3062, 3035, 2862, 1674, 1597, 1627, 1492, 1091, 1064, 756, 698; ^1^H-NMR (200 MHz, CD_3_OD) δ 7.36 (m, 10H), 4.68 (m, 2H), 3.17 (dt, 1H, *J* = 16.0, 2.0 Hz), 2.76 (dt, 1H, *J* = 14.0, 2.0 Hz), 2.51 (dd, 1H, *J* = 14.0, 12.0 Hz), 2.27 (dd, *J* = 14.0, 12.0 Hz, 1H); ^13^C-NMR (50 MHz, CD_3_OD) δ 159.01, 158.57, 144.10, 130.80, 130.24, 128.20, 82.20, 44.71, 38.37; Anal. Calcd for C_18_H_21_ClN_4_O C, 62.69; H, 6.14; N, 16.25; Found C, 62.27, H, 6.24, N, 16.15.

*2-(2,6-Bis(4-fluorophenyl)-2H-pyran-4(3H)-ylidene)hydrazinecarboximidamida* (**3**). This product was obtained using 0.5 mmol of 2,6-bis(4-fluorophenyl)-tetrahydropyran-4-one (**8b**). The product was purified by column chromatography on silica gel using methanol/EtOAc (1:9) as eluent, resulting in quantitative yield (100%). IR (KBr) ν/cm^−1^ 3390, 3352, 3271, 3066, 2958, 2850, 1670, 1604, 1627, 1508, 1080, 1056, 825. ^1^H-NMR (200 MHz, CD_3_OD) δ 7.45 (dd, 4H, *J* 14.0, 8.0), 7.04 (t, 4H, *J* = 8.0 Hz), 4.63 (t, 2H, *J* = 12.0 Hz), 2.72 (d, 1H, *J* = 16.0 Hz), 2.48 (dd, 1H, *J* = 14.0, 12.0 Hz), 2.22 (dd, 1H, *J* = 14.0, 12.0 Hz), 2.08 (s, 1H); ^13^C-NMR (50 MHz, CD_3_OD) δ 167.43, 162.56, 159.86, 157.84, 140.17, 130.29, 117.19, 81.64, 44.65, 38.24; Anal. Calcd for C_18_H_19_ClF_2_N_4_O C, 56.77; H, 5.03; N, 14.71; Found C, 56.63, H, 5.06, N, 14.77.

*2-(2,6-Di(naphthalen-2-yl)-2H-pyran-4(3H)-ylidene)hydrazinecarboximidamida* (**4**). This product was obtained using 0.5 mmol of 2,6-di(naphthalen-2-yl)-tetrahydropyran-4-one (**8c**). The product was purified by column chromatography on silica gel using methanol/EtOAc (3:7) as eluent, resulting in quantitative yield (100%). IR (KBr) ν/cm^−1^ 3423, 3340, 3155, 3055, 1672, 1600, 1625, 1508, 1078, 817, 748. ^1^H-NMR (200 MHz, CD_3_OD) δ 7.87 (m, 8H), 7.60 (m, 2H), 7.43 (m, 4H), 4.78 (dd, 2H, *J* = 12.0, 2.0 Hz), 2.69 (m, 4H); ^13^C-NMR (50 MHz, CD_3_OD) δ 158.98, 158.34, 141.44, 135.97, 135.83, 130.43, 130.04, 128.55, 128.41, 127.13, 126.90, 126.35, 82.24, 44.49, 38.26; Anal. Calcd for C_26_H_24_N_4_O C, 76.45; H, 5.92; N, 13.72; Found C, 76.55, H, 6.01, N, 13.64.

#### 3.1.6. General Procedure for the Preparation of Aminoguanidine (**5**–**7**)

The aminoguanidine were prepared by reduction of guanylhydrazone with sodium cyanoborohydride. The guanylhydrazone 0.5 mmol was added to a dry flask under magnetic stirring, followed by addition of 1 mL of ethanol at 0 °C. Then, 48 mg of sodium cyanoborohydride was slowly added to the flask, and then the mixture remained stirring at room temperature for two hours. After completion of the reaction, the solvent was evaporated under reduced pressure. This crude product was then subjected to flash column chromatography to yield a solid.

*2-(2,6-Diphenyltetrahydro-2H-pyran-4-yl)hydrazinecarboximidamida* (**5**). This product was obtained using 0.5 mmol of 2-(2,6-diphenyl-2*H*-pyran-4-(3*H*)-ylidene) hydrazinecarboximidamida (**2**). The product was purified by column chromatography on silica gel using EtOAc as the eluent, resulting in a yield of 94%. IR (KBr) ν/cm^−1^ 3568, 3448, 3062, 3032, 2862, 1674, 1631, 1631, 1492, 1126, 1064, 756, 698; ^1^H-NMR (200 MHz, CD_3_OD) δ 7.33 (m, 10H), 5.40 (s, 1H), 4.60 (m, 2H), 3.10 (d, 1H, *J* = 14.6 Hz), 2.33 (m, 2H), 1.92 (m, 2H), 1.16 (t, 2H, *J* = 8.0 Hz); ^13^C-NMR (50 MHz, CD_3_OD) δ 161.81, 159.32, 158.24, 145.58, 145.09, 144.08, 143.32, 130.76, 130.54, 128.19, 82.02, 80.46, 44.71, 42.66, 40.69, 38.21; Anal. Calcd for C_18_H_23_ClN_4_O C, 62.33; H, 6.68; N, 16.15; Found C, 62.38, H, 6.64, N, 16.19.

*2-(2,6-Bis-(4-fluorophenyl)tetrahydro-2H-pyran-4-yl)hydrazinecarboximidamida* (**6**). This product was obtained using 0.5 mmol of 2-(2,6-bis(4-fluorophenyl)-2*H*-pyran-4(3*H*)-ylidene)-hydrazinecarboximidamida (**3**). The product was purified by column chromatography on silica gel using EtOAc as eluent, resulting in quantitative yield (100%). IR (KBr) ν/cm^−1^ 3568, 3452, 2924, 2870, 1666, 1639, 1639, 1512, 1126, 1076, 833; ^1^H-NMR (200 MHz, CD_3_OD) δ 7.47 (m, 4H), 7.07 (m, 4H), 4.7 (m, 2H), 3.53 (m, 2H), 1.61 (m, 8H); ^13^C-NMR (50 MHz, CD_3_OD) δ 168.48, 163.63, 159.25, 142.88, 142.82, 142.44, 142.38, 131.49, 118.63, 118.21, 81.27, 77.61, 41.92, 39.37. Anal. Calcd for C_18_H_20_F_2_N_4_O C, 62.42; H, 5.82; N, 16.18; Found C, 62.48, H, 5.79, N, 16.16.

*2-(2,6-Di(naphthalen-2-yl)tetrahydro-2H-pyran-4-yl)hydrazinecarboximidamida* (**7**). This product was obtained using 0.5 mmol of 2-(2,6-di(naphthalen-2-yl)-2*H*-pyran-4(3*H*)-ylidene)hydrazinecarboximidamida (**4**). The product was purified by column chromatography on silica gel using EtOAc as eluent, resulting in quantitative yield (100%). IR (KBr) ν/cm^-1^ 3525, 3448, 3055, 2943, 1674, 1631, 1124, 1070, 819, 750; ^1^H-NMR (200 MHz, CD_3_OD) δ 7.69 (m, 14H), 4.79 (s, 2H), 2.44 (m, 5H), 1.22 (m, 2H); ^13^C-NMR (50 MHz, CD_3_OD) δ 163.17, 160.56, 159.60, 144.35, 143.83, 142.76, 142.04, 137.29, 137.14, 136.96, 131.64, 131.58, 131.23, 129.78, 129.66, 129.40, 128.47, 128.40, 128.20, 128.01, 127.93, 127.67, 83.64, 82.10, 78.42, 60.92, , 45.87, 41.97, 39.44; Anal. Calcd for C_26_H_27_ClN_4_O C, 69.87; H, 6.09; N, 12.53; Found C, 69.83, H, 6.12, N, 12.64.

### 3.2. Pharmacology

Fetal bovine serum (FBS) (Cripion, Brazil); Ficoll-Hypaque (GE Healthcare, Uppsala, Sweden); Trypan blue (Inlab, São Paulo, Brazil); trypsin–EDTA and MTT (3-(4,5-Dimethylthiazol-2-yl)-2,5-diphenyl tetrazolium bromide) (Amresco, Toronto, ON, Canada); RPMI1640 and DMEM medium was purchased from HIMEDIA (Mumbai, Maharashtra, India) and Penicillin/streptomycin solution was obtained by Sigma-Aldrich (St. Louis, MO, USA).

#### 3.2.1. Preparation of Stock Solutions

Preparation of stock solutions of the test compounds were prepared in dimethyl sulfoxide (DMSO) at 20 mmol·L^−1^, filtered through adequate filters (0.22 µm), and diluted for use in the nutrient medium to the relevant working concentrations. For all of the cells used, the nutrient medium was RPMI 1640 or DMEM, according to the cell line tested. We did not verify any DMSO effect, incubating only this dispersing at the highest concentration used in the experiments.

#### 3.2.2. Cell Culture

K562, HL-60, MCF-7, HT-29 and L929 cell lines were acquired from Rio de Janeiro Cell Bank (Federal University of Rio de Janeiro, Rio de Janeiro, RJ, Brazil). Leukemia cell lines and MCF-7 cells were maintained in the nutrient RPMI 1640 medium supplemented with 10% FBS, 2 mmol·L^−1^ glutamine, 100 U·mL^−1^ penicillin, and 100 µg·mL^−1^ streptomycin. Others cell lines were maintained in the nutrient DMEM similarly supplemented. All of these cells were grown at 37 °C in 5% CO_2_ and humidified air atmosphere. The PBMC cells were separated by Histopaque-1077 (GE Healthcare), from whole blood of healthy, non-smoking donors who had not taken any drugs for at least 15 days prior to sampling donors. Thus, using the gradient centrifugation, the interface cells were washed with PBS (phosphate buffered saline), counted and resuspended in nutrient medium to the required cell concentration. The blood used to obtain PBMC cells were collected from the blood bank of the João Pessoa (Hemocentro), Paraíba, Brazil. Similar procedure was performed for obtaining PBMC/CML. These cells were collected from blood of patients recently diagnosed with CML and without being subject to treatment at this moment. The blood samples from patients PBMC/CML, were collected in the Napoleão Laureano Hospital, João Pessoa, Paraíba, Brazil. This study was approved by the Institutional Ethical Committee of Lauro Wanderley Hospital (CEP/HULW), from Federal University of Paraíba, protocol numbers, 378119 and 05878712.7.0000.5183.

#### 3.2.3. Cell Viability–MTT

The cytotoxicity of molecules to cells was evaluated using the original enzymatic reduction of 3-(4,5-dimethylthiazol-2-yl)-2,5-diphenyltetrazolium bromide (MTT) assay to produce formazan crystals [33]. Cells were seeded at 3 × 10^4^ or 5 × 10^4^ cells per well in 96-well tissue culture plates. Cells were exposed to different concentrations of tetrahydropyrans substituted (3–100 µmol·L^−1^) dissolved in the medium (three wells per concentration). After 24 h or 72 h of incubation, plates were centrifuged (500 g, 5 min) and the supernatant was removed, followed by the addition of MTT solution (0.5 mg·mL^−1^ in PBS) and incubation for 4 h at 37 °C. After 4 h, the MTT formazan product was dissolved in SDS/HCl 0.01 mol·L^−1^ and absorbance was measured at 570 nm in reader plate (Biotek, Winooski, VT, USA).

## 4. Conclusions

In this paper we present efficient syntheses of six new guanylhydrazone and aminoguanidine tetrahydropyran derivatives **2**–**7**. We studied the cytotoxic activity of studied tetraydropyran compounds (**2**–**7**) in various cancer and normal cell lines; in K562 cells the cytotoxicity is mediated by cell-cycle arrest in the G1 phase. These results can address a promising starting point for furthering structural modifications in these compounds as a new class of anticancer compounds, leading to the possibility of target-directed drug design for cancer treatment.

## Figures and Tables

**Table 1:** Inhibitory effect of compounds and etoposide on cell growth of human cancer and normal cell lines after 24 h of incubation.

Compound	HL-60	K562	HT-29	MCF-7	LMC	L929	PBMC
**2**	16.0 ± 4.6	13.6 ± 4.5	15.9 ± 3.7	52.0 ± 4.6	nt	18.1 ± 5.0	19.4 ± 3.2
**3**	12.5 ± 4.5	8.9 ± 4.0	11.2 ± 4.0	31.7 ± 4.7	7.5 ± 2.4	20.0 ± 4.2	14.3 ± 3.2
**4**	9.2 ± 4.6	7.5 ± 4.9	7.3 ± 4.7	16.7 ± 5.2	nt	10.8 ± 5.5	5.9 ± 3.3
**5**	42.0 ± 4.4	44.7 ± 4.8	46.9 ± 4.2	>100	nt	65.8 ± 4.7	51.5 ± 3.0
**6**	85.4 ± 5.2	58.8 ± 4.1	59.5 ± 4.3	126.9 ± 4.3	nt	72.2 ± 3.9	72.7 ± 3.4
**7**	35.3 ± 5.2	15.5 ± 5.1	25.8 ± 4.8	43.3 ± 5.8	14.8 ± 2.4	39.7 ± 5.5	20.8 ± 3.5
**Etoposide**	5.8 ± 1.1	>50	>100	>100	nt	nt	>100

Note: nt (not tested); PBMC, peripheral blood mononuclear cell; SEM, standard error of the mean. HL-60: leukemia myeloid acute, K562: leukemia myeloid chronic, MCF-7: breast adenocarcinoma cell line, HT-29: colon adenocarcinoma cell line and PBMC were incubated with compounds for 24 h separately. The cell viability was determined by MTT assay. Results are displayed as IC_50_ in micromolar values and expressed as the mean ± SEM, obtained from at least three independent experiments in triplicate.

**Table 2:** Inhibitory effect of compounds and etoposide on cell growth of human cancer and normal cell lines after 72 h of incubation.

Compound	HL-60	K562	HT-29	MCF-7	PBMCLMC	L929	PBMC
**2**	14.4 ± 5.2	31.0 ± 4.5	9.6 ± 4.7	34.8 ± 3.8	nt	23.9 ± 4.0	4.7 ± 2.9
**3**	14.9 ± 5.4	29.0 ± 4.9	6.0 ± 4.6	20.8 ± 4.0	14.6 ± 3.4	17.2 ± 4.7	6.7 ± 3.1
**4**	6.9 ± 4.9	19.2 ± 5.3	3.4 ± 4.7	13.7 ± 5.0	nt	3.9 ± 3.8	2.2 ± 2.9
**5**	41.4 ± 5.8	71.4 ± 5.0	19.9 ± 3.9	47.2 ± 3.5	nt	46.9 ± 4.4	11.4 ± 2.7
**6**	66.5 ± 5.3	>100	36.9 ± 3.3	42.9 ± 4.2	nt	79.4 ± 3.8	12.5 ± 2.9
**7**	25.4 ± 5.3	43.6 ± 5.6	11.8 ± 4.3	24.14 ± 3.5	18.7 ± 2.7	16.88 ± 4.1	7.5 ± 3.4

Note: nt (not tested); PBMC, peripheral blood mononuclear cell; SEM, standard error of the mean. HL-60: leukemia myeloid acute, K562: leukemia myeloid chronic, MCF-7: breast adenocarcinoma cell line, HT-29: colon adenocarcinoma cell line and PBMC were incubated with compounds for 24 h separately. The cell viability was determined by MTT assay. Results are displayed as IC_50_ in micromolar values and expressed as the mean ± SEM, obtained from at least three independent experiments in triplicate.

## Supplementary Materials

Supplementary materials can be accessed at: http://www.mdpi.com/1420-3049/21/6/671/s1.
