# Supplementary material for: Synthesis and Anticancer Activities of Novel Guanylhydrazone and Aminoguanidine Tetrahydropyran Derivatives
Source: Molecules. 2016 Jun 21;21(6):671. doi: 10.3390/molecules21060671 (PMC6274535; doi:10.3390/molecules21060671)
Supplement: Supplementary file 1 [file molecules-21-00671-s001.pdf]

Molecules **2016**, *21*, 671; doi:10.3390/molecules21060671

Supplementary Materials: Synthesis and Anticancer Activities of Novel Guanylhydrazone and Aminoguanidine Tetrahydropyran Derivatives

Fábio Pedrosa Lins Silva, Bruna Braga Dantas, Gláucia Veríssimo Faheina Martins, Demétrius Antônio Machado de Araújo and Mário Luiz Araújo de Almeida Vasconcellos

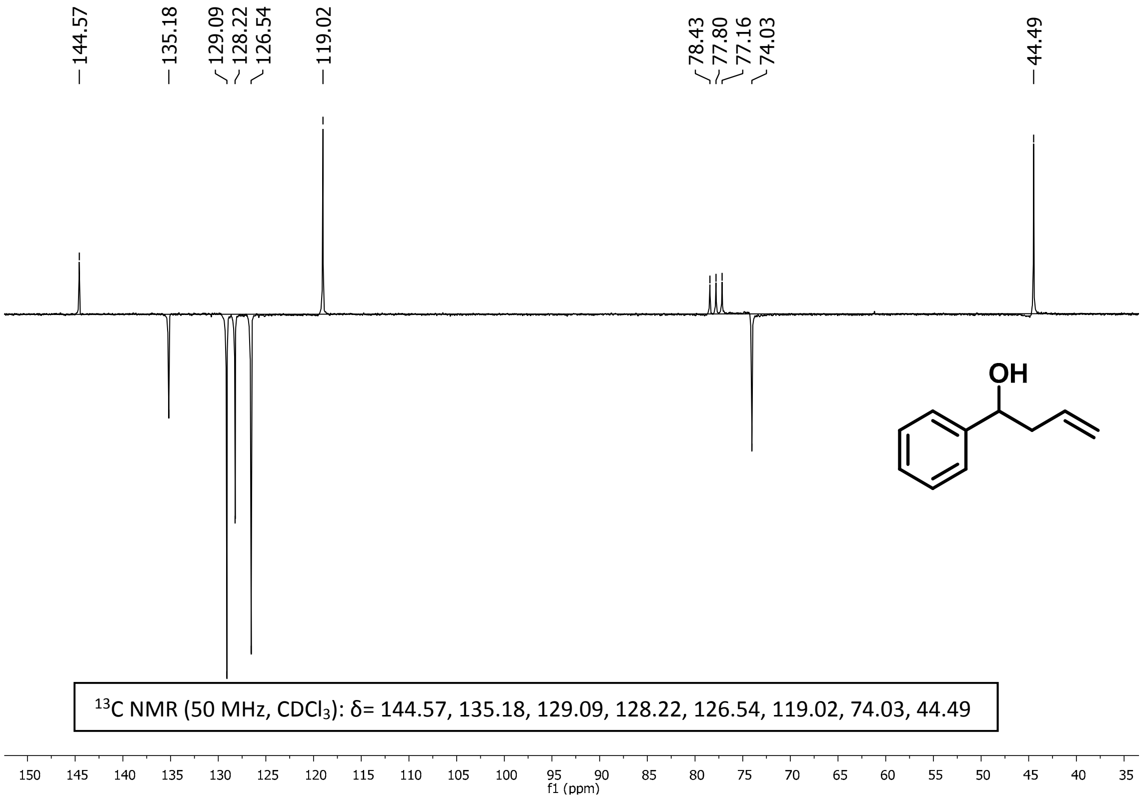

Figure S1. ^{13}C NMR-APT $CDCl_3$, 50 MHz) of 1-phenylbut-3-en-1-ol.

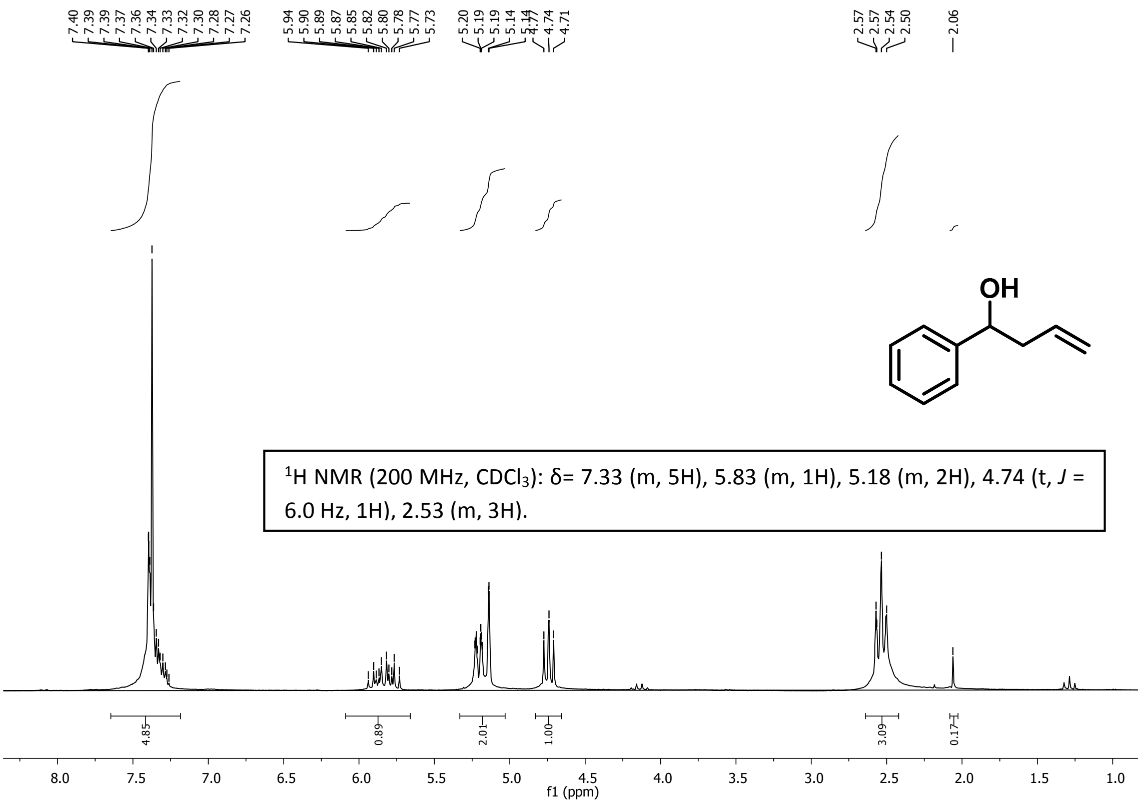

Figure S2. ^{1}H NMR (CDCl$_3$, 200 MHz) of 1-phenylbut-3-en-1-ol.

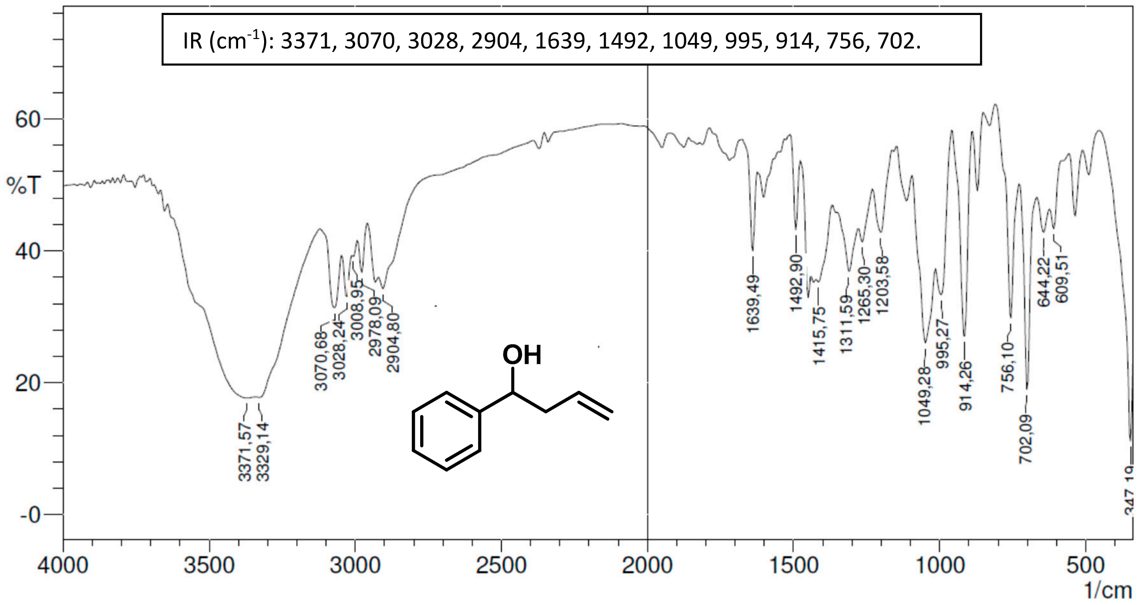

Figure S3. IR (KBr, cm^{-1}) of 1-phenylbut-3-en-1-ol.

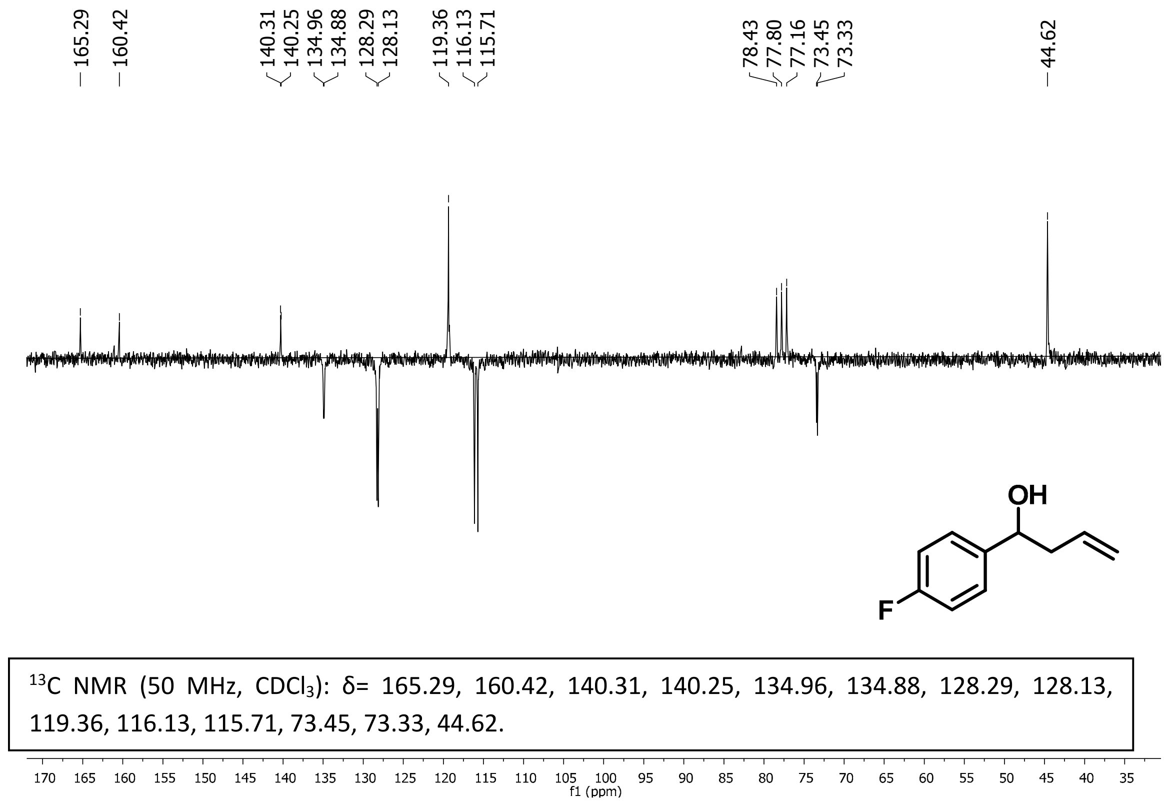

Figure S4. NMR ^{13}C-APT (CDCl$_3$, 50 MHz) of 1-(4-fluorophenyl)but-3-en-1-ol.

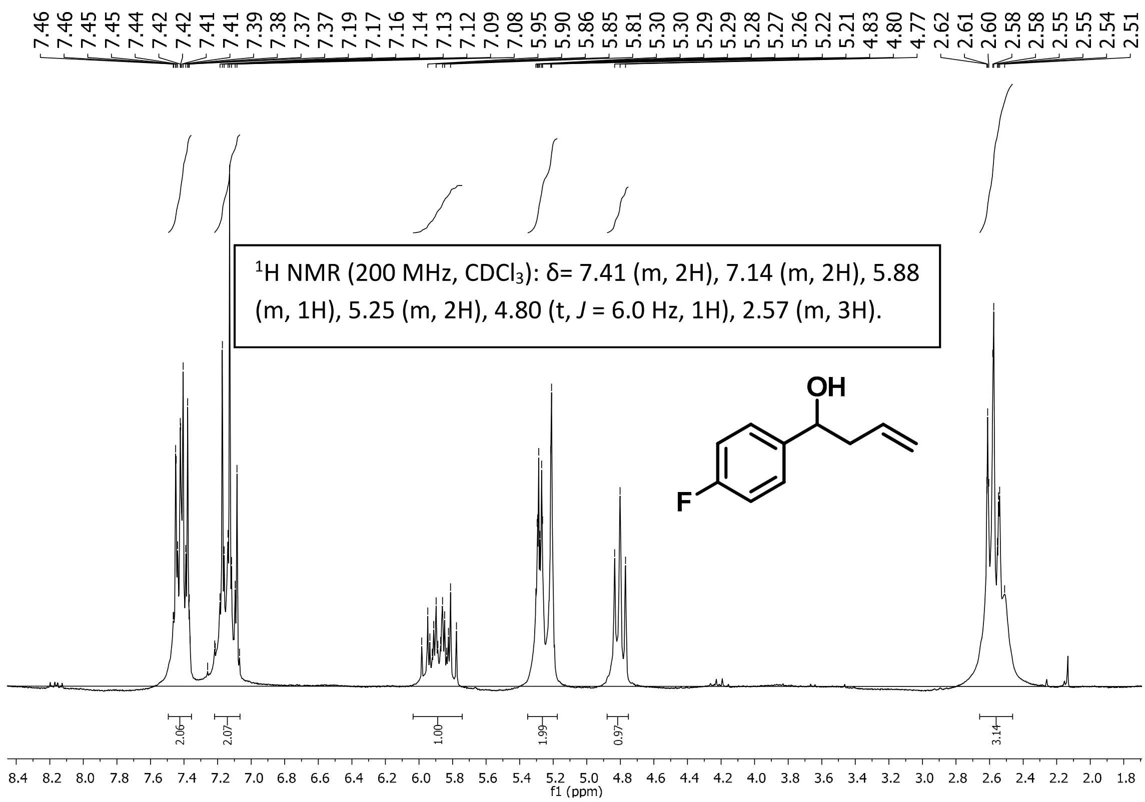

Figure S5. ^{1}H NMR (CDCl$_3$, 200 MHz) of 1-(4-fluorophenyl)but-3-en-1-ol.

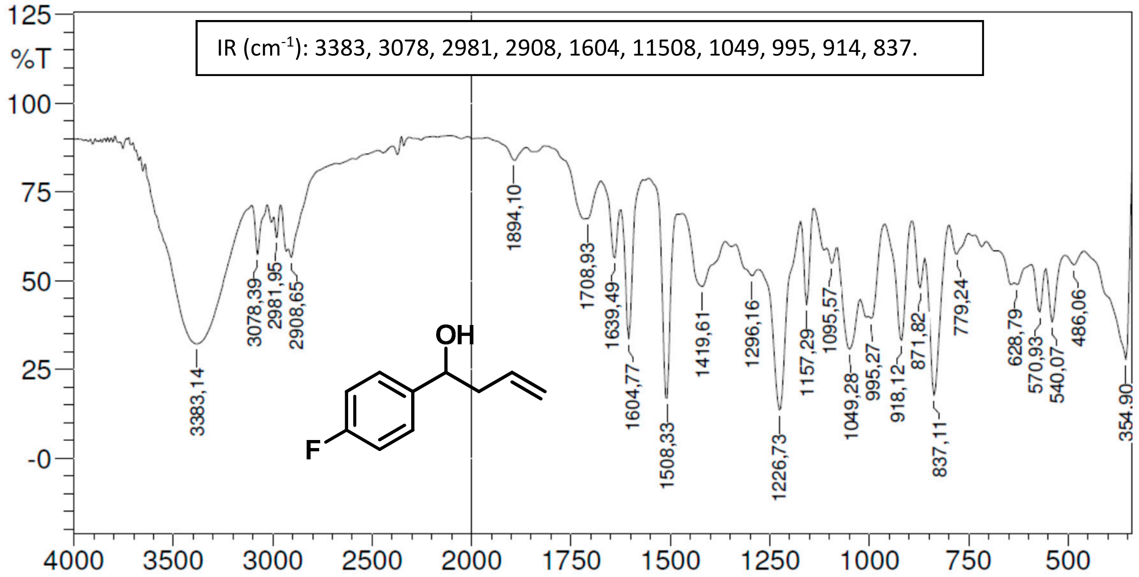

Figure S6. IR (KBr, cm-1) of 1-(4-fluorophenyl)but-3-en-1-ol.

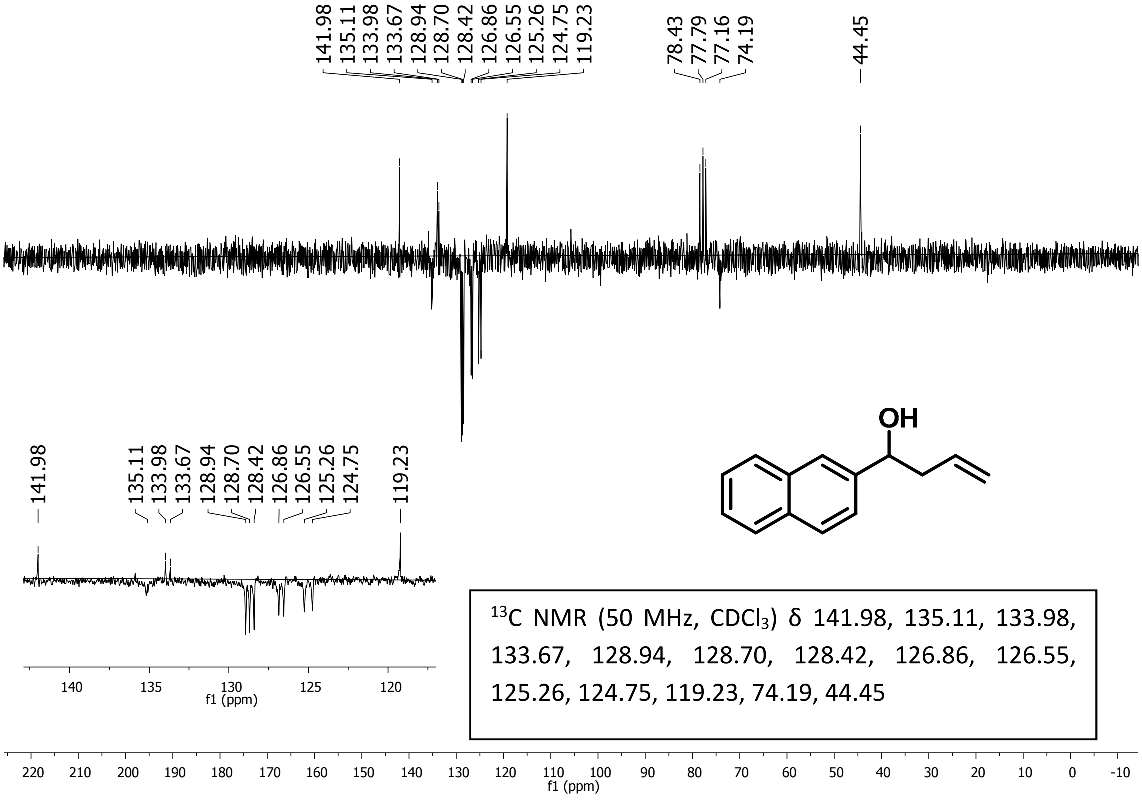

Figure S7. ^{13}C NMR-APT ($CDCl_3$, 50 MHz) of 1-(naphthalen-2-yl)but-3-en-1-ol.

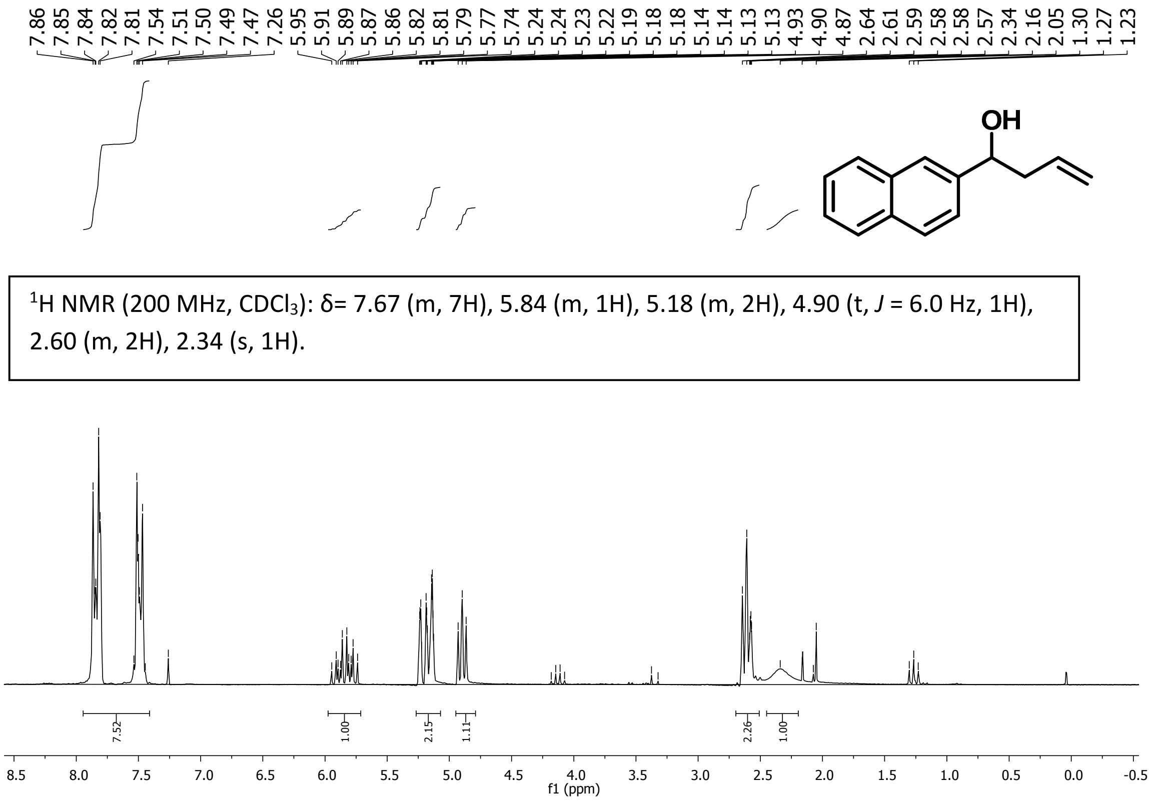

Figure S8. ^1H NMR ($CDCl_3$, 200 MHz) of 1-(naphthalen-2-yl)but-3-en-1-ol.

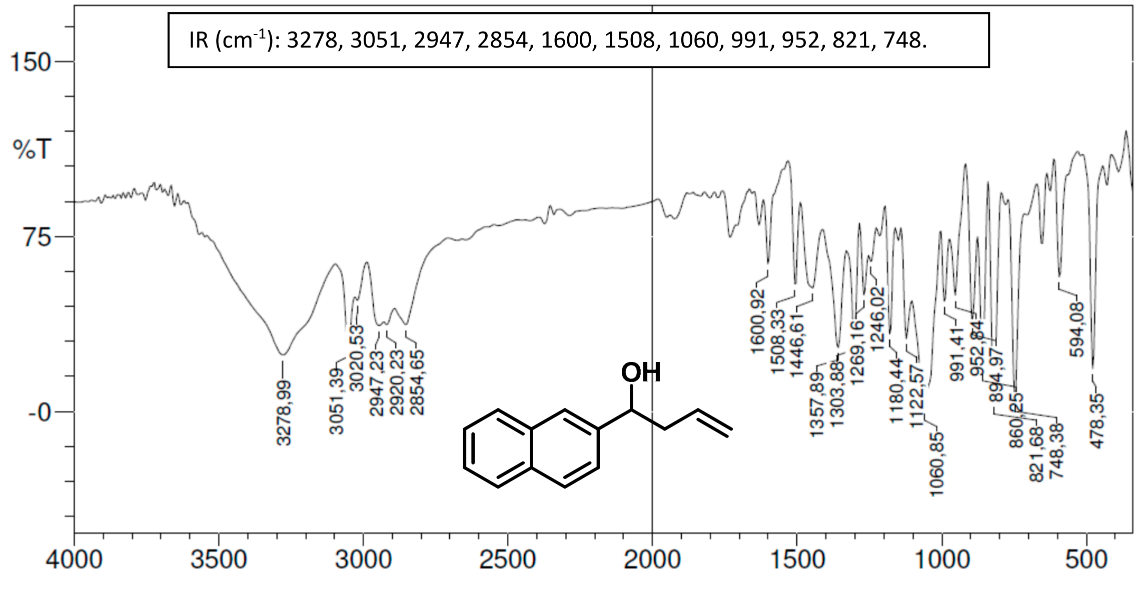

Figure S9. IR (KBr, cm^{-1}) of 1-(naphthalen-2-yl)but-3-en-1-ol.

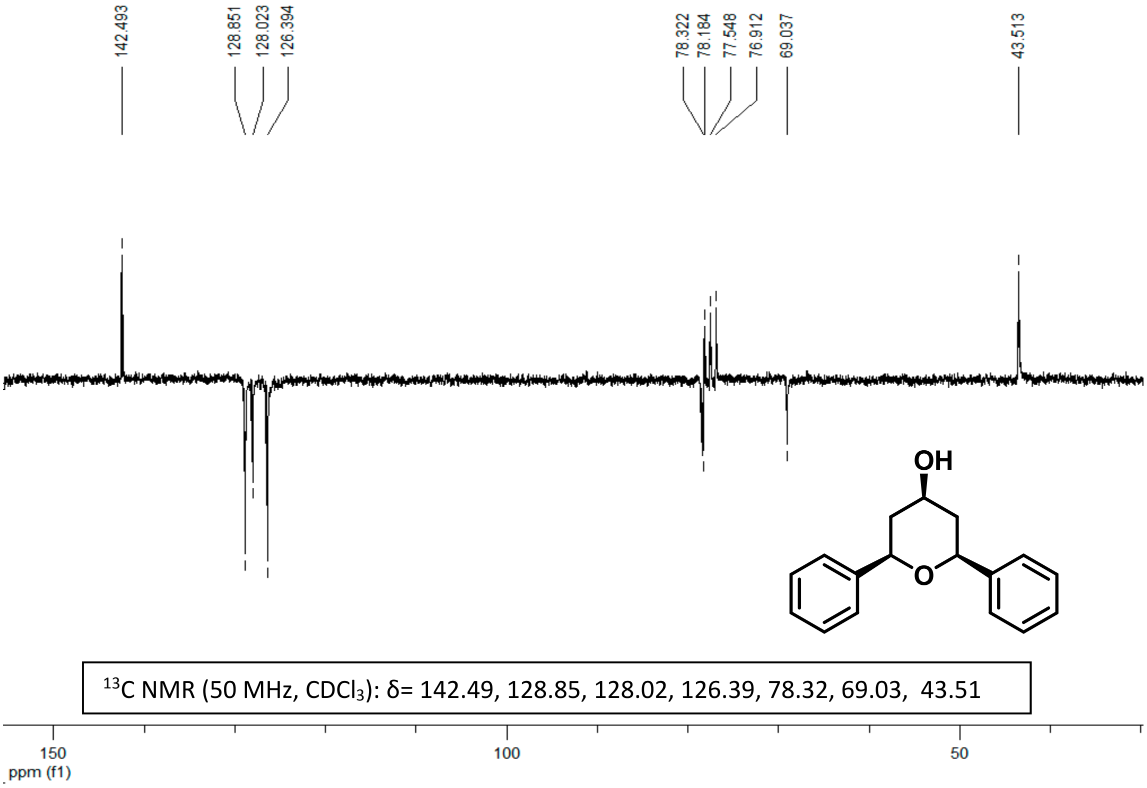

Figure S10. ^{13}C NMR-APT ($CDCl_3$, 50 MHz) of 2,6-diphenyl-tetrahydro-2*H*-pyran-4-ol (**1a**).

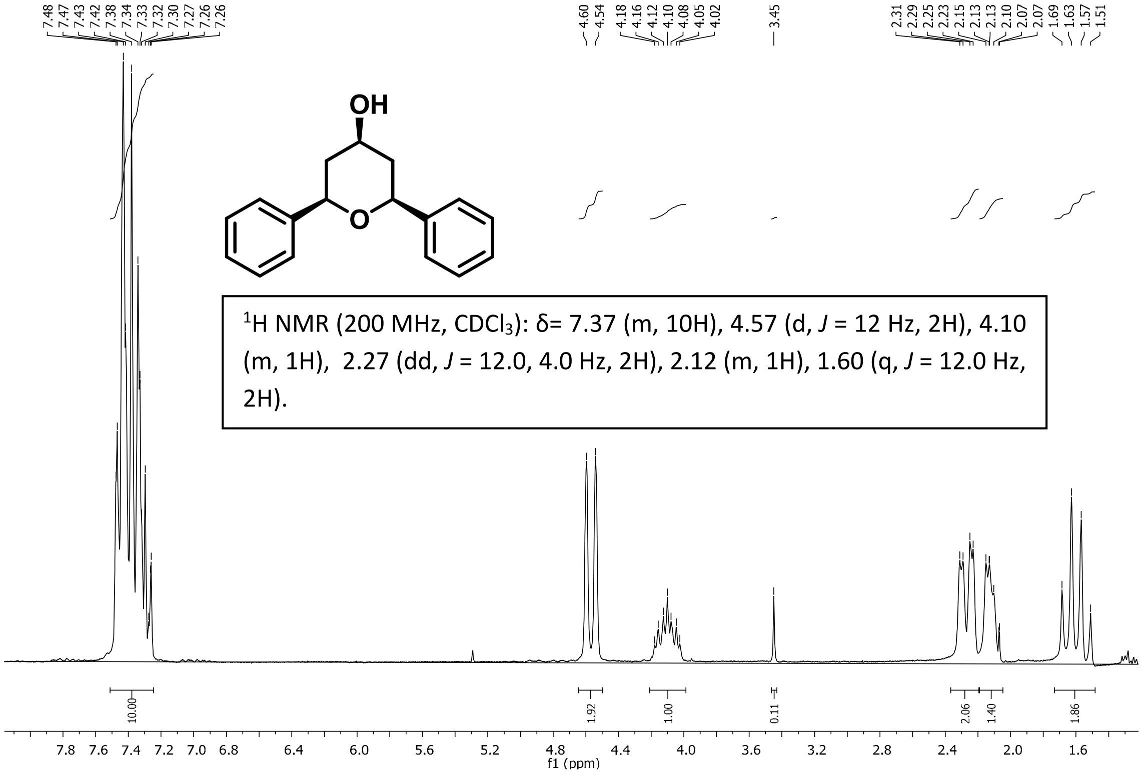

Figure S11. ^{1}H NMR ($CDCl_3$, 200 MHz) of 2,6-diphenyl-tetrahydro-2*H*-pyran-4-ol (**1a**).

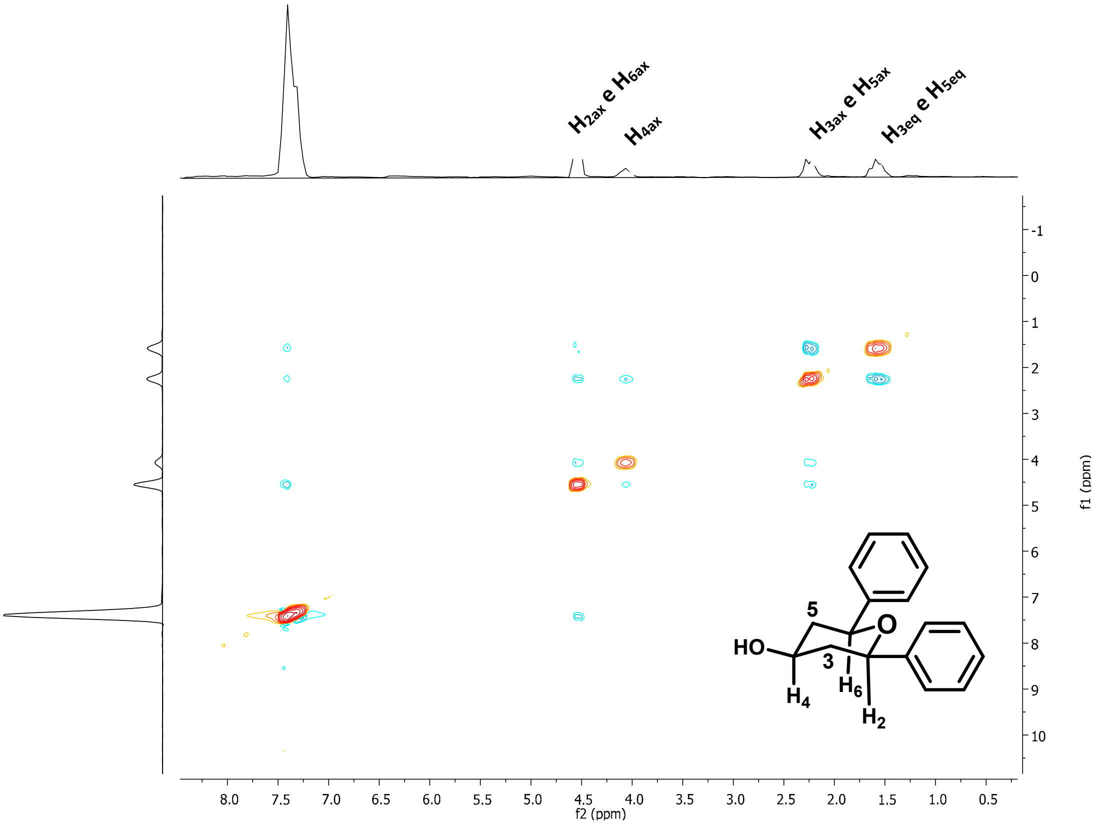

Figure S12. NOSY of 2,6-diphenyl-tetrahydro-2*H*-pyran-4-ol (**1a**).

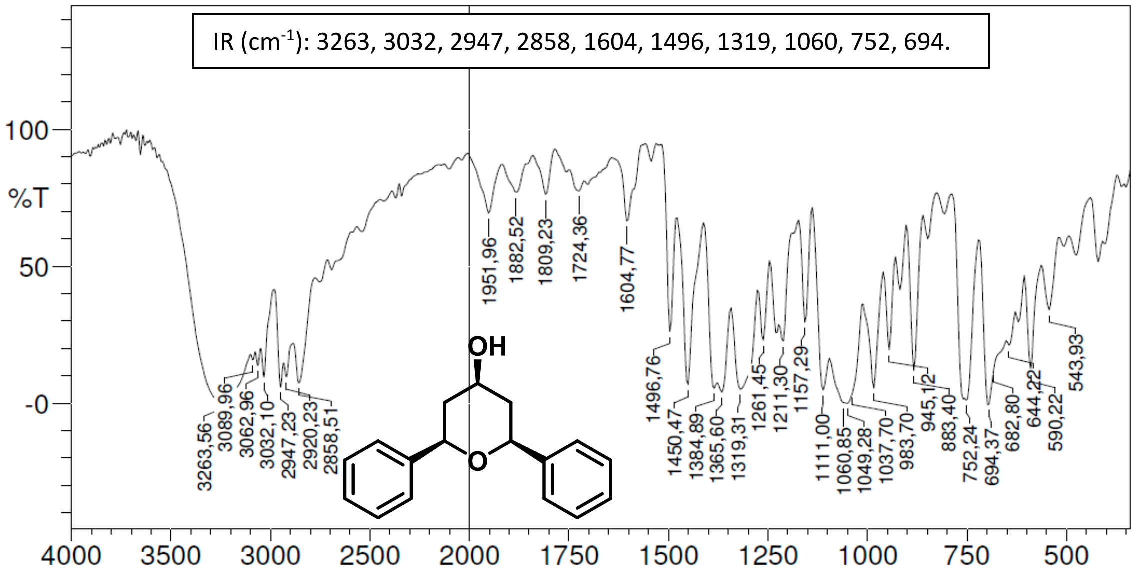

Figure S13. IR (KBr, cm^{-1}) of 2,6-diphenyl-tetrahydro-2*H*-pyran-4-ol (**1a**).

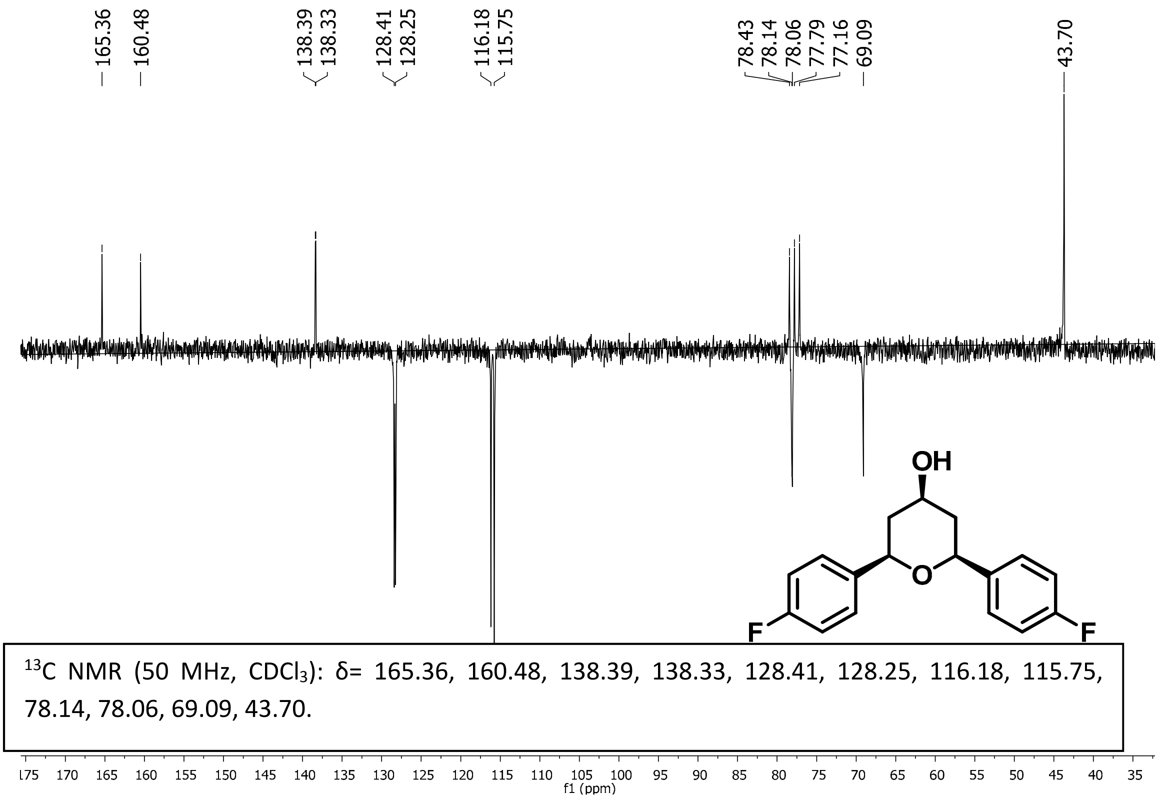

Figure S14. ^{13}C NMR-APT ($CDCl_3$, 50 MHz) of 2,6-di(naphthalen-2-yl)-tetrahydro-2*H*-pyran-4-ol (**1b**).

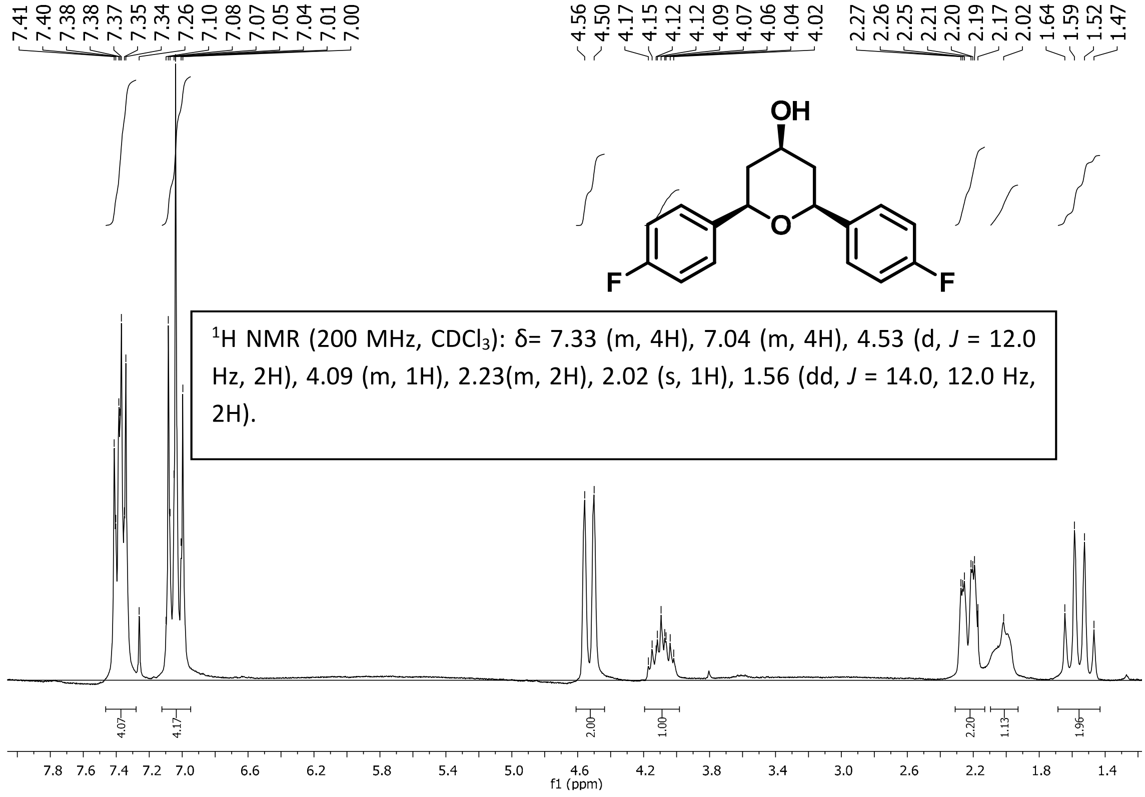

Figure S15. ^{1}H NMR ($CDCl_3$, 200 MHz) of 2,6-di(naphthalen-2-yl)-tetrahydro-2*H*-pyran-4-ol (**1b**).

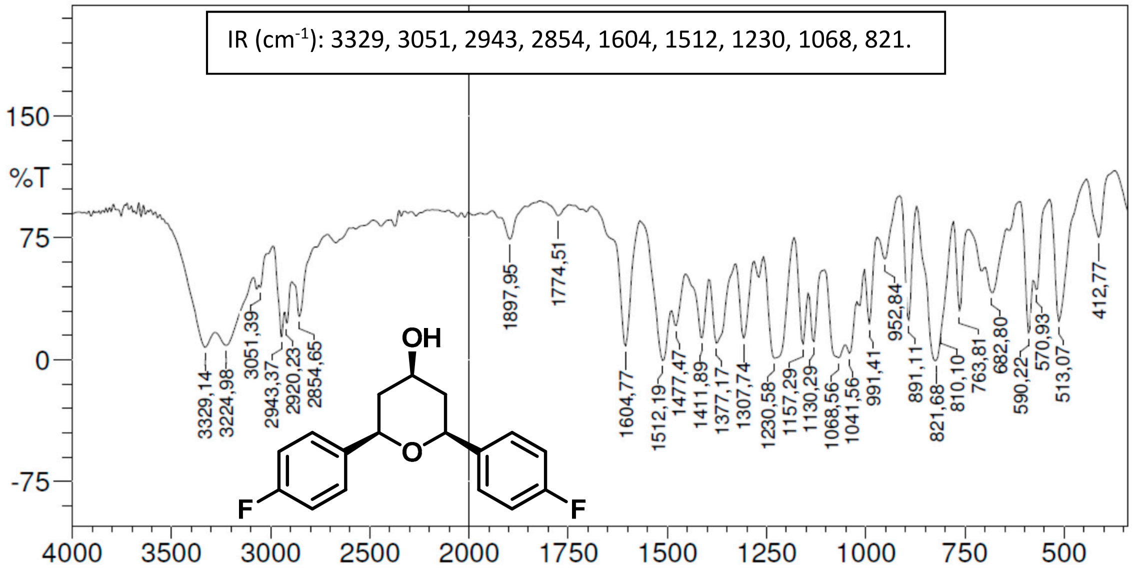

Figure S16. IR (KBr, cm^{-1}) of 2,6-di(naphthalen-2-yl)-tetrahydro-2*H*-pyran-4-ol (**1b**).

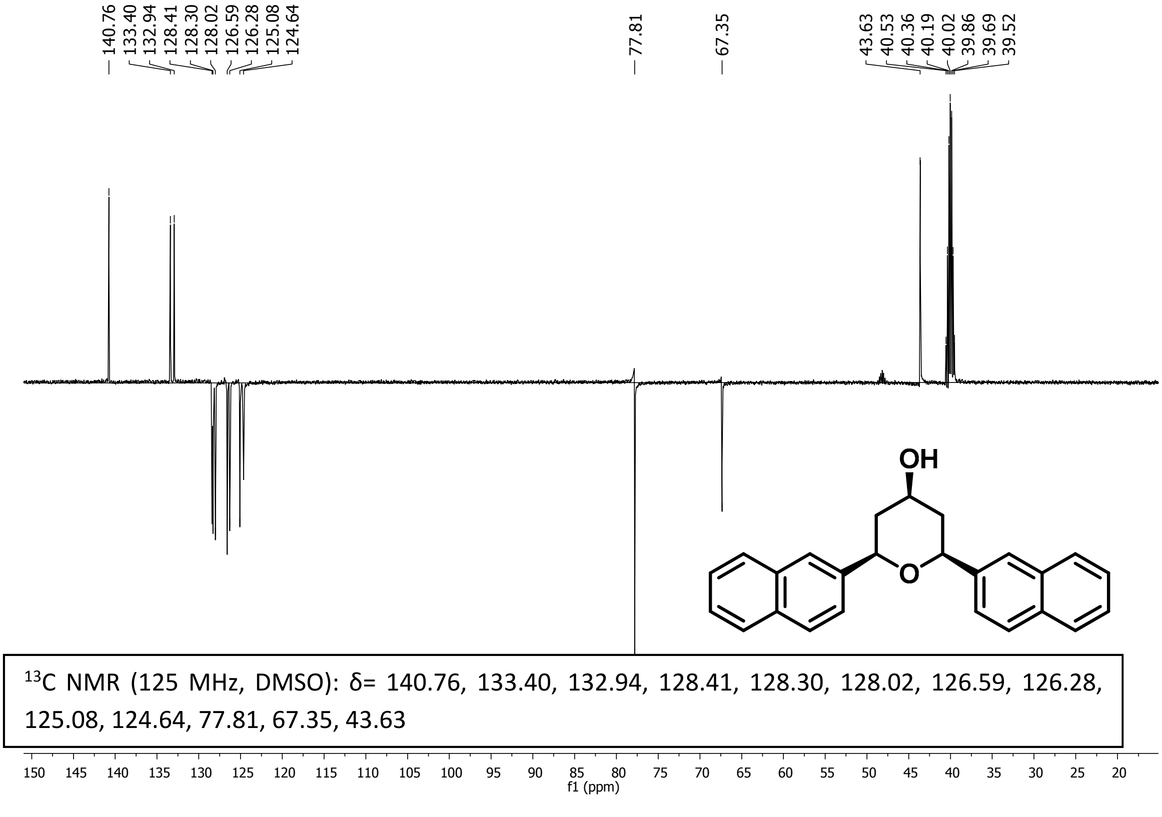

Figure S17. ^{13}C NMR-APT (DMSO, 125 MHz) of 2,6-di(naphthalen-2-yl)-tetrahydro-2*H*-pyran-4-ol (**1c**).

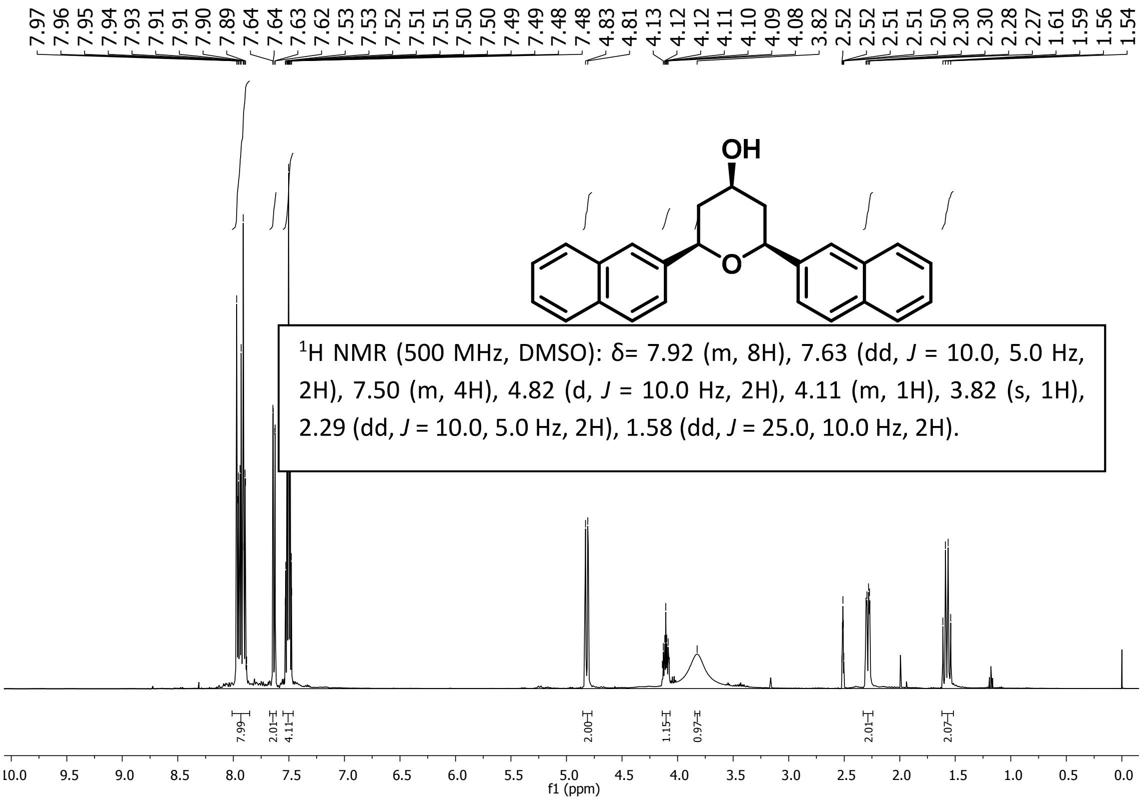

Figure S18. ^{1}H NMR (DMSO, 500 MHz) of 2,6-di(naphthalen-2-yl)-tetrahydro-2*H*-pyran-4-ol (**1c**).

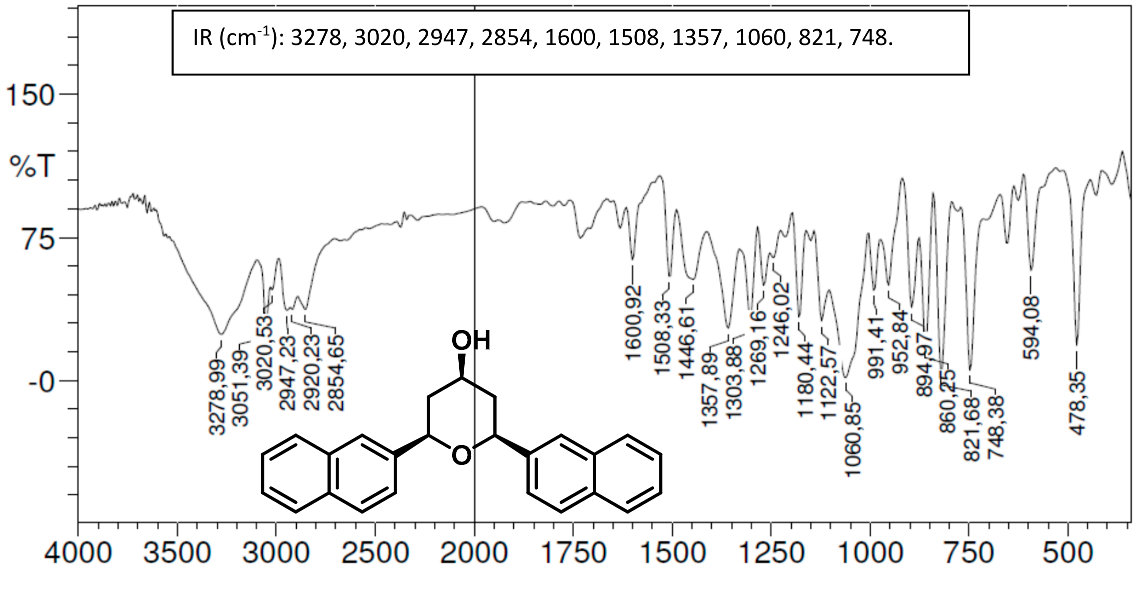

Figure S19. IR (KBr, cm^{-1}) of 2,6-di(naphthalen-2-yl)-tetrahydro-2*H*-pyran-4-ol (**1c**).

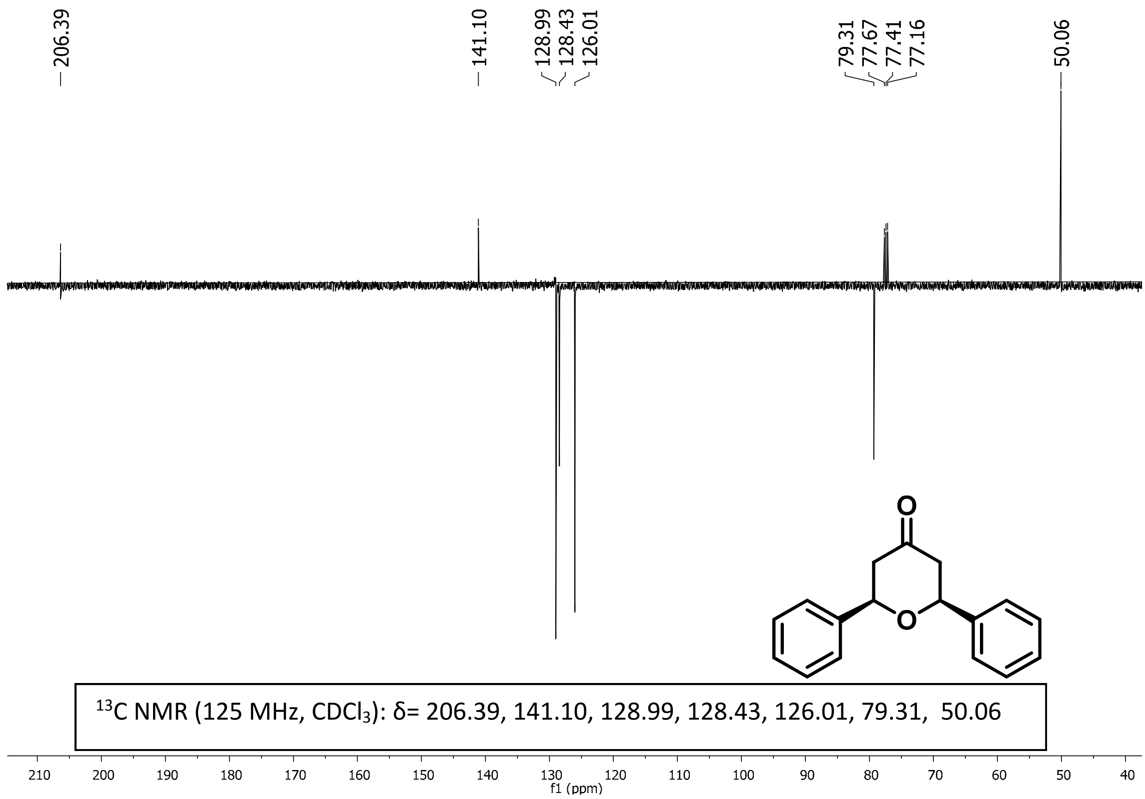

Figure S20. ^{13}C NMR-APT ($CDCl_3$, 125 MHz) of 2,6-diphenyl-tetrahydropyran-4-one (**8a**).

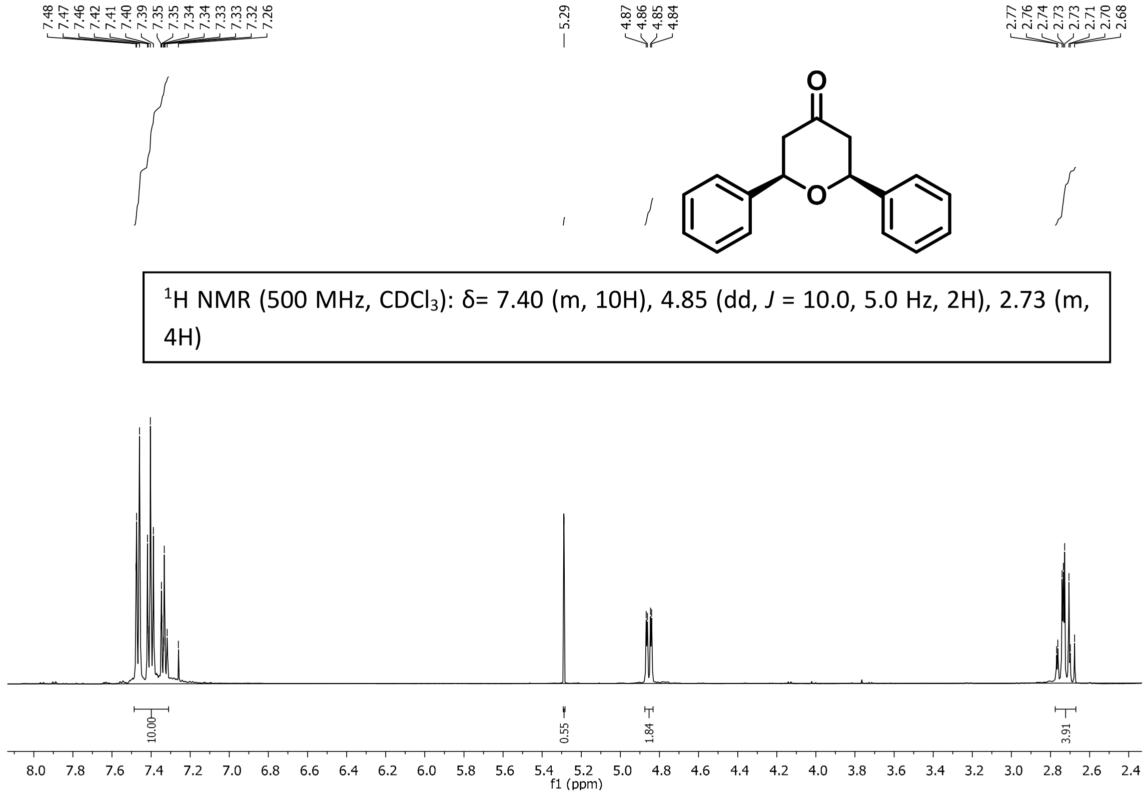

Figure S21. ^{1}H NMR ($CDCl_3$, 500 MHz) of 2,6-diphenyl-tetrahydropyran-4-one (**8a**).

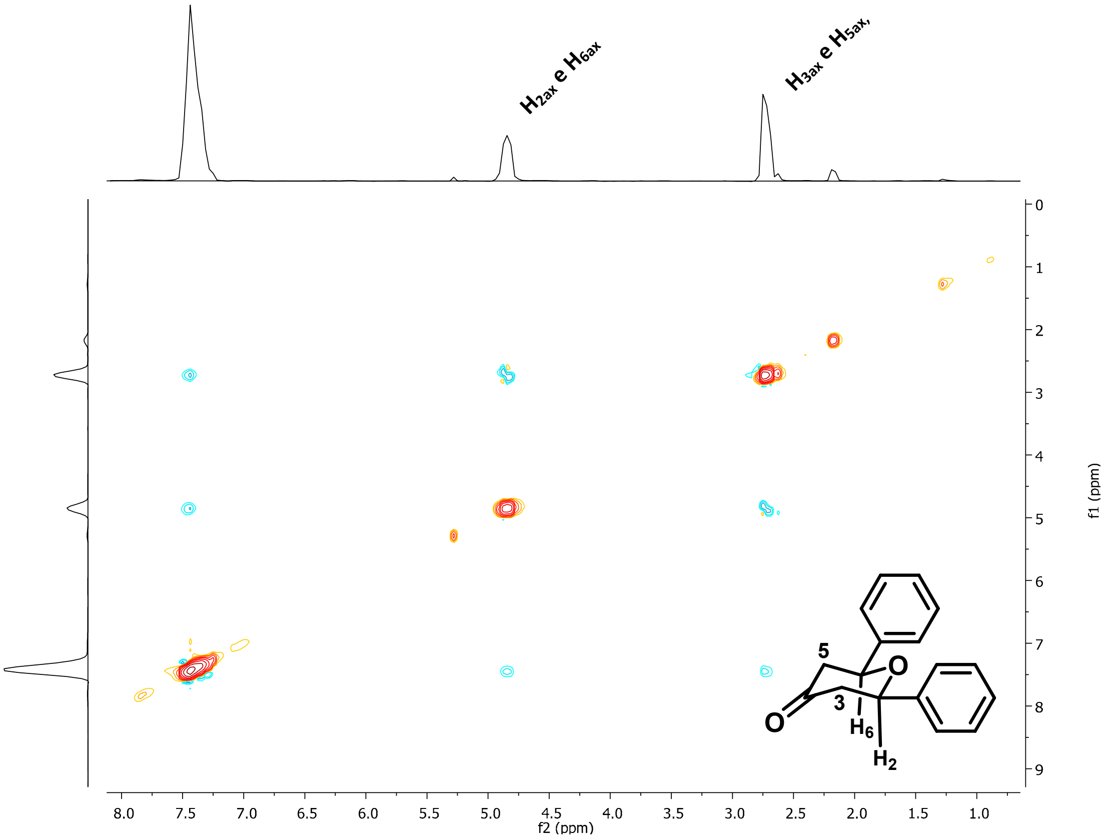

Figure S22. NOSY of 2,6-diphenyl-tetrahydropyran-4-one (**8a**).

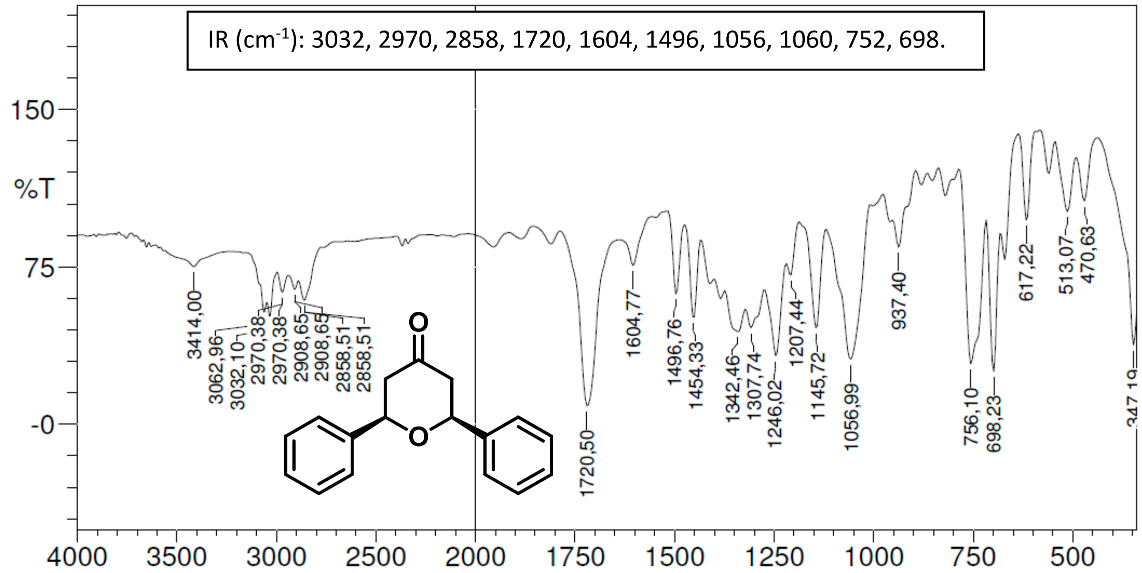

Figure S23. IR (KBr, cm^{-1}) of 2,6-diphenyl-tetrahydropyran-4-one (**8a**).

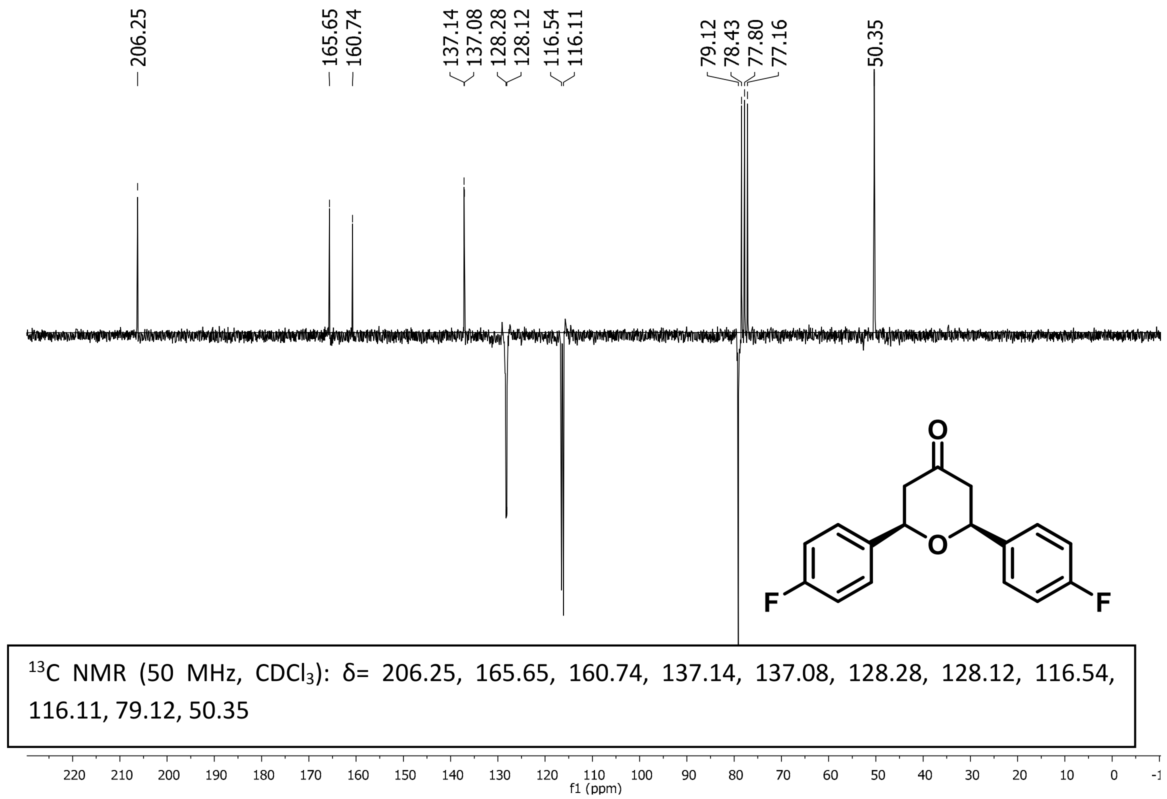

Figure S24. ^{13}C NMR-APT (CDCl$_3$, 50 MHz) of 2,6-bis(4-fluorophenyl)-tetrahydropyran-4-one (**8b**).

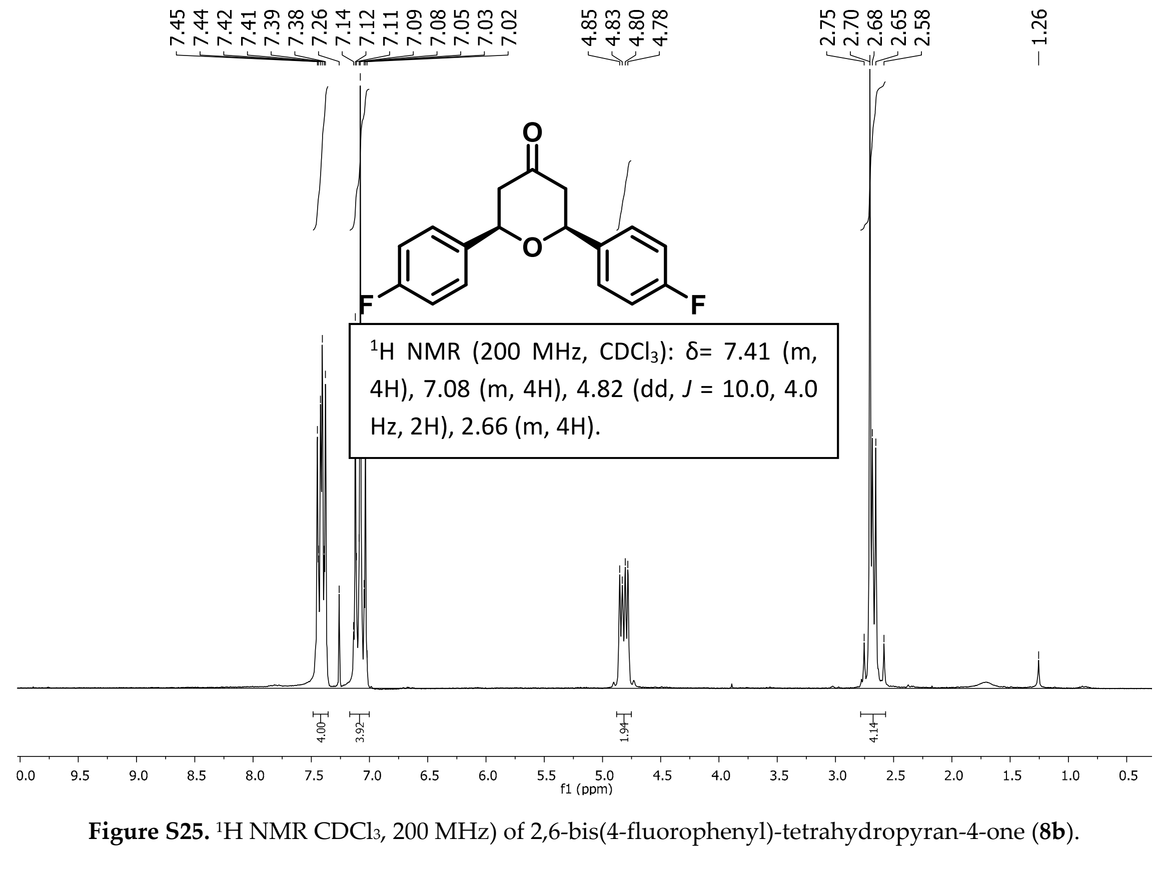

Figure S25. ^{1}H NMR CDCl$_3$, 200 MHz) of 2,6-bis(4-fluorophenyl)-tetrahydropyran-4-one (**8b**).

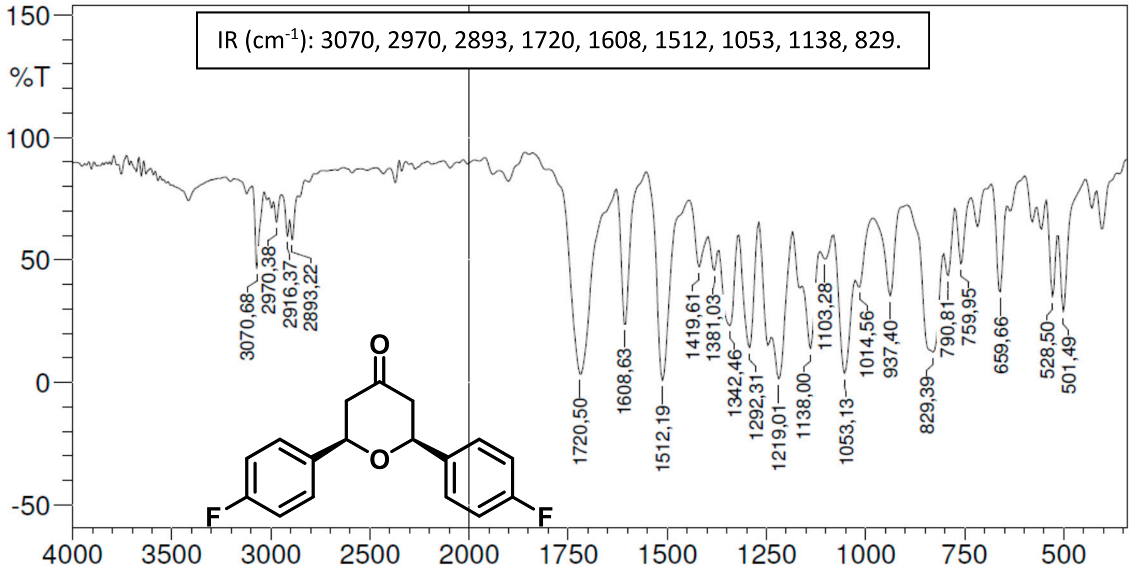

Figure S26. IR (KBr, cm^{-1}) of 2,6-bis(4-fluorophenyl)-tetrahydropyran-4-one (**8b**).

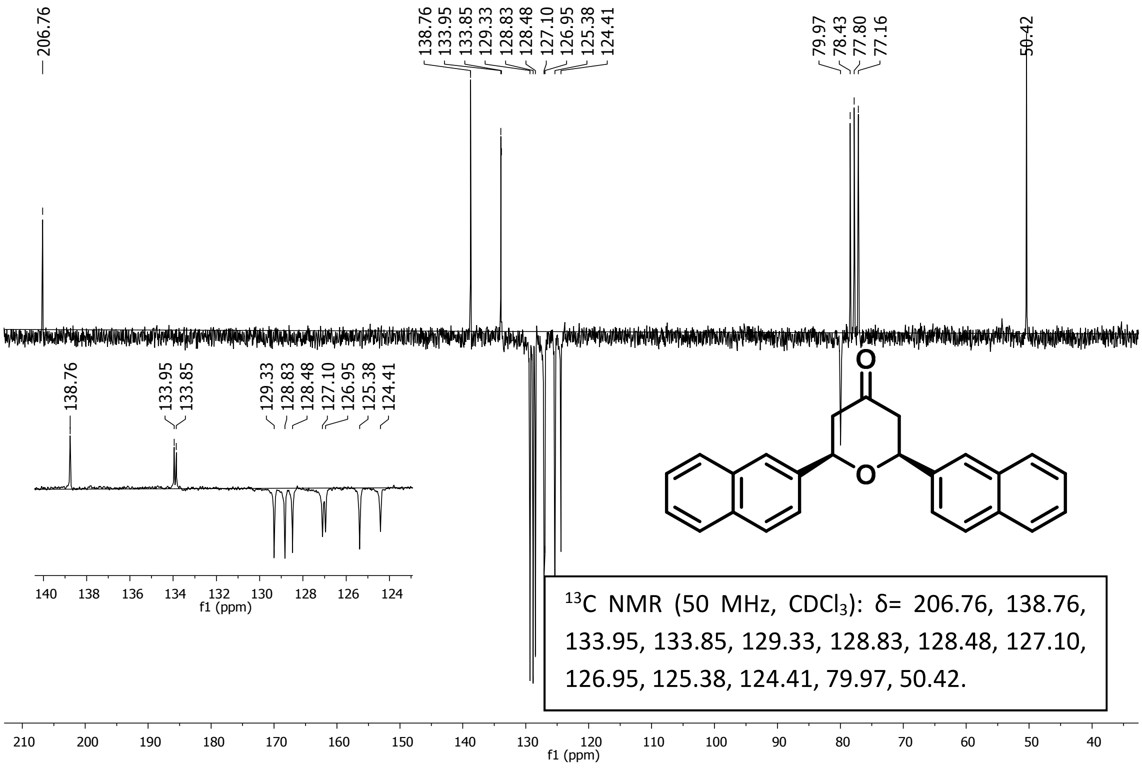

Figure S27. ^{13}C NMR-APT ($CDCl_3$, 50 MHz) of 2,6-di(naphthalen-2-yl)-tetrahydropyran-4-one (**8c**).

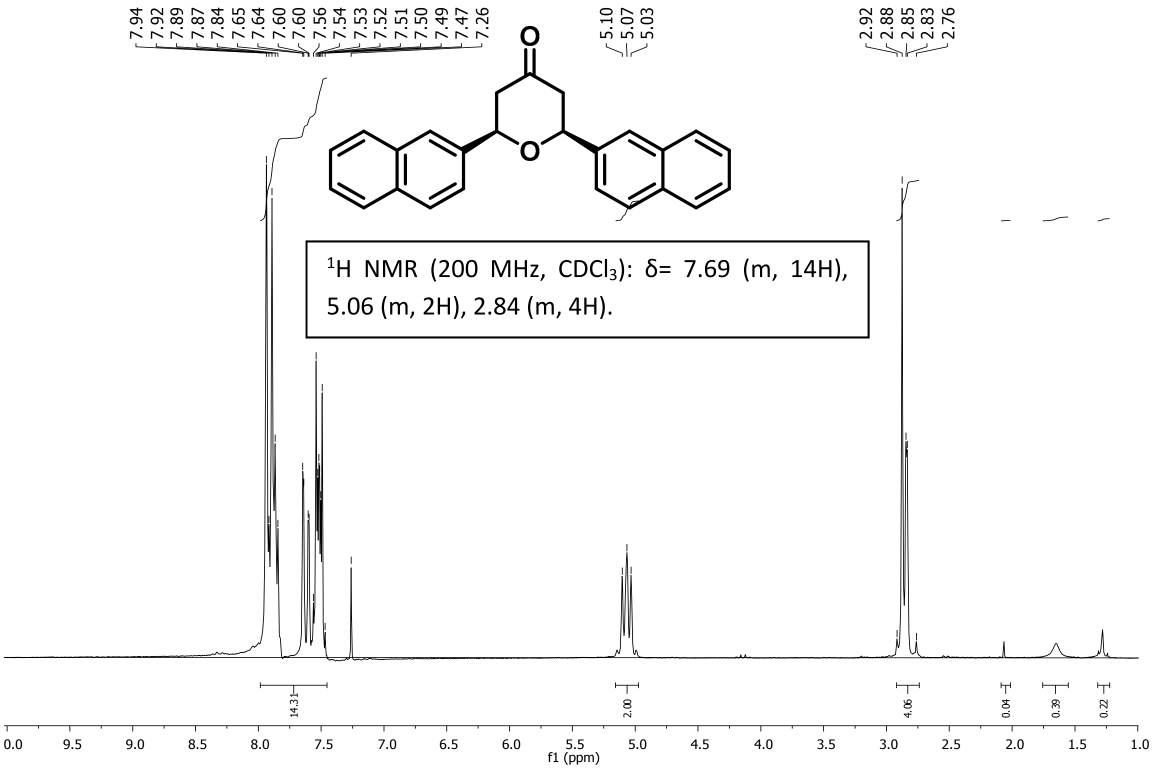

Figure S28. ^{1}H NMR (CDCl$_3$, 200 MHz) of 2,6-di(naphthalen-2-yl)-tetrahydropyran-4-one (**8c**).

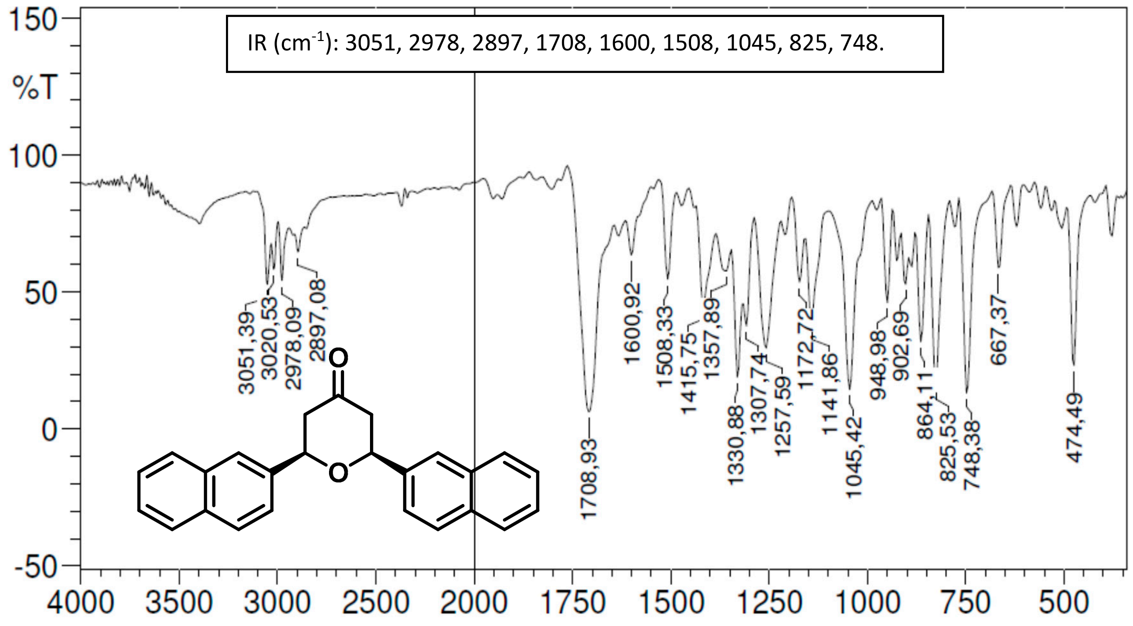

Figure S29. IR (KBr, cm^{-1}) of 2,6-di(naphthalen-2-yl)-tetrahydropyran-4-one (**8c**).

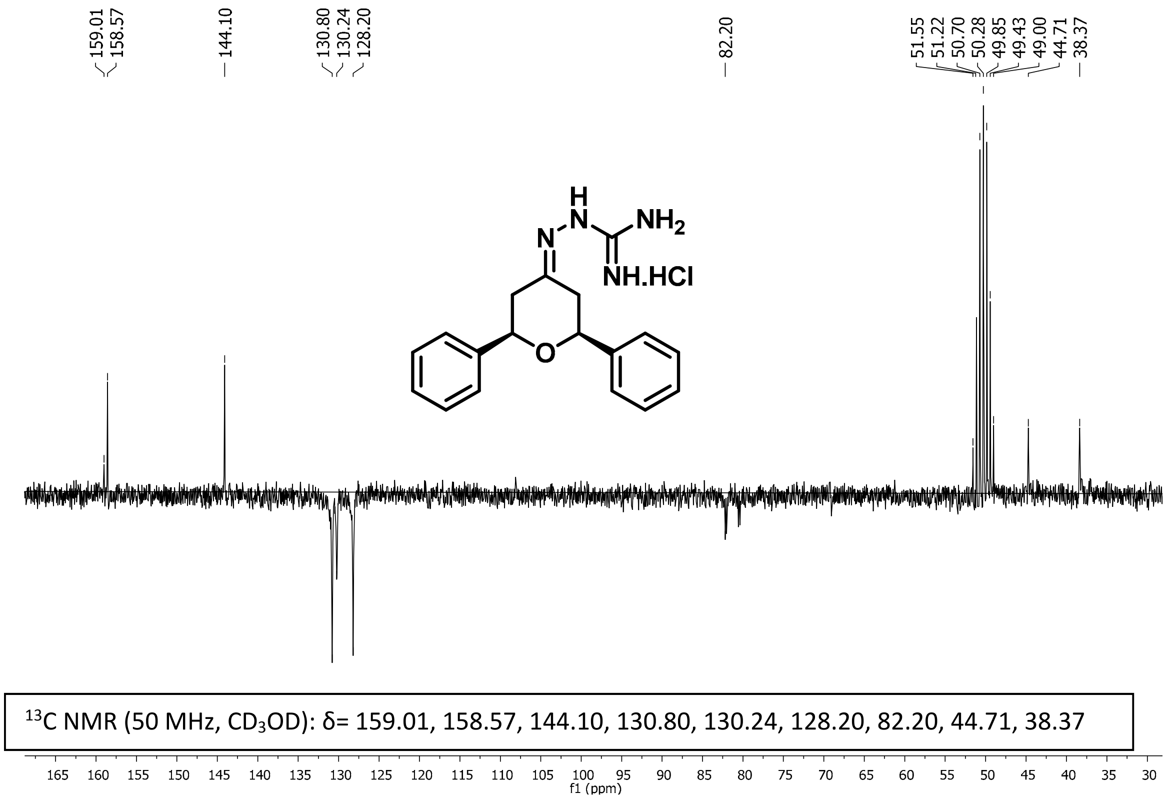

Figure S30. ^{13}C NMR-APT (CD_3OD, 50 MHz) of 2-((cis-2,6-diphenyldihydro-2H-pyran-4(3H)-ylidene)hydrazinecarboximidamide hydrochloride (**2**).

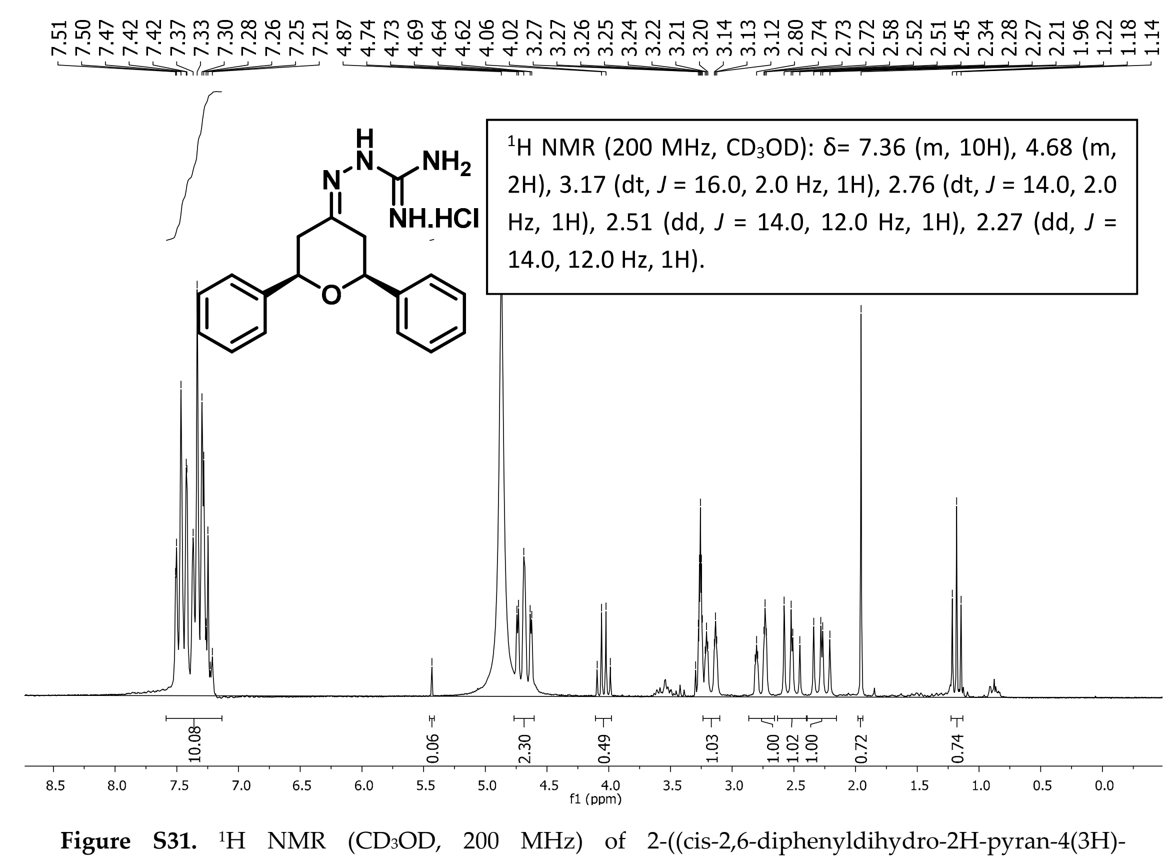

Figure S31. ^{1}H NMR (CD_3OD, 200 MHz) of 2-((cis-2,6-diphenyldihydro-2H-pyran-4(3H)-ylidene)hydrazinecarboximidamide hydrochloride (**2**).

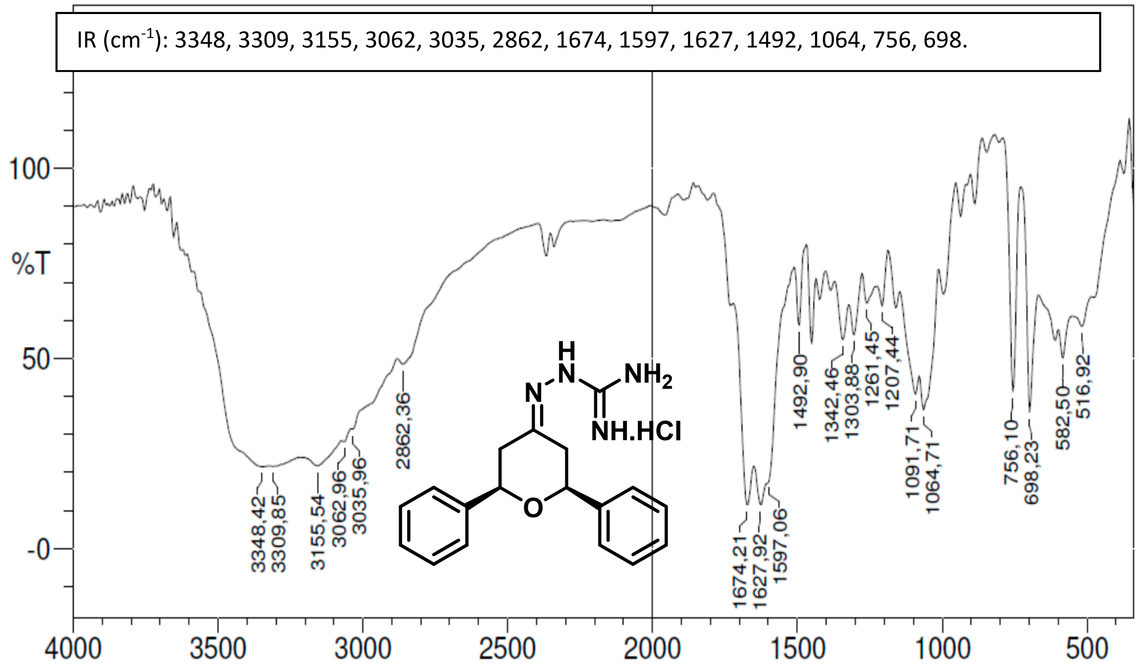

Figure S32. IR (KBr, cm-1) 2-((cis-2,6-diphenyldihydro-2H-pyran-4(3H)-ylidene)hydrazinecarboximidamide hydrochloride (**2**).

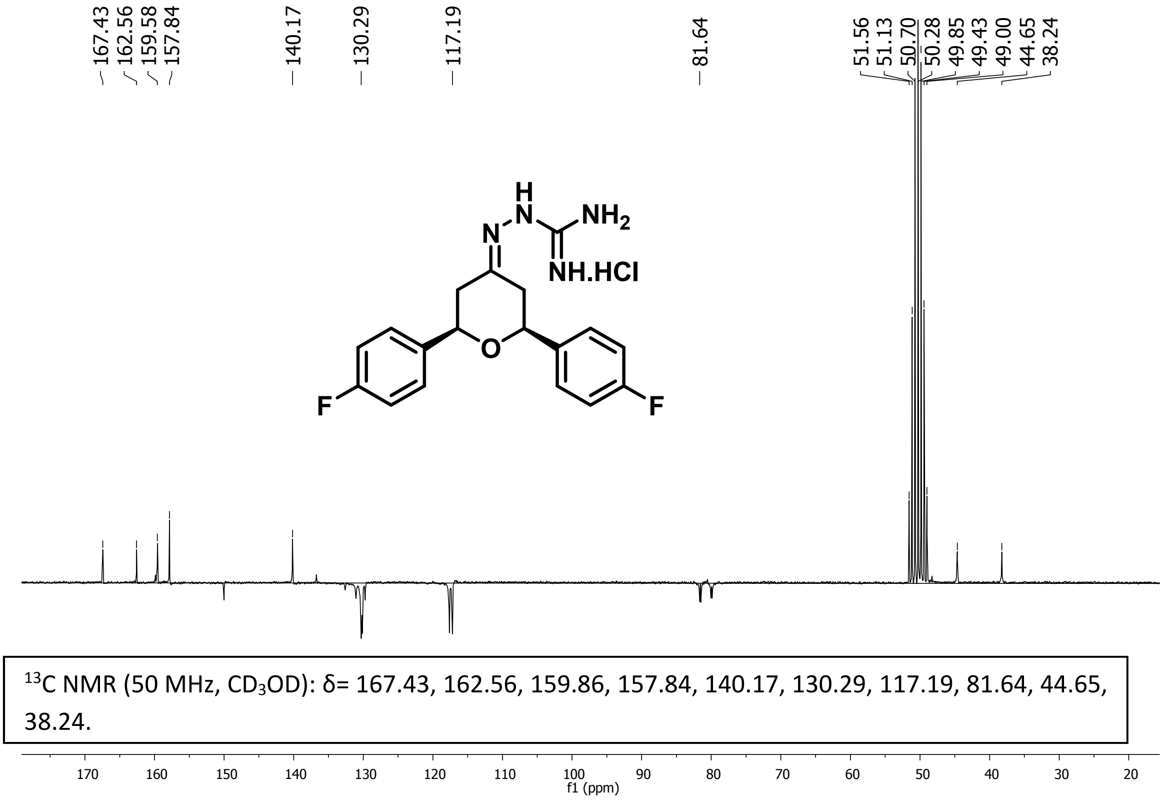

Figure S33. ^{13}C NMR-APT (CD_3OD, 50 MHz) of 2-((cis-2,6-bis(4-fluorophenyl)dihydro-2H-pyran-4(3H)-ylidene)hydrazinecarboximidamide hydrochloride (**3**).

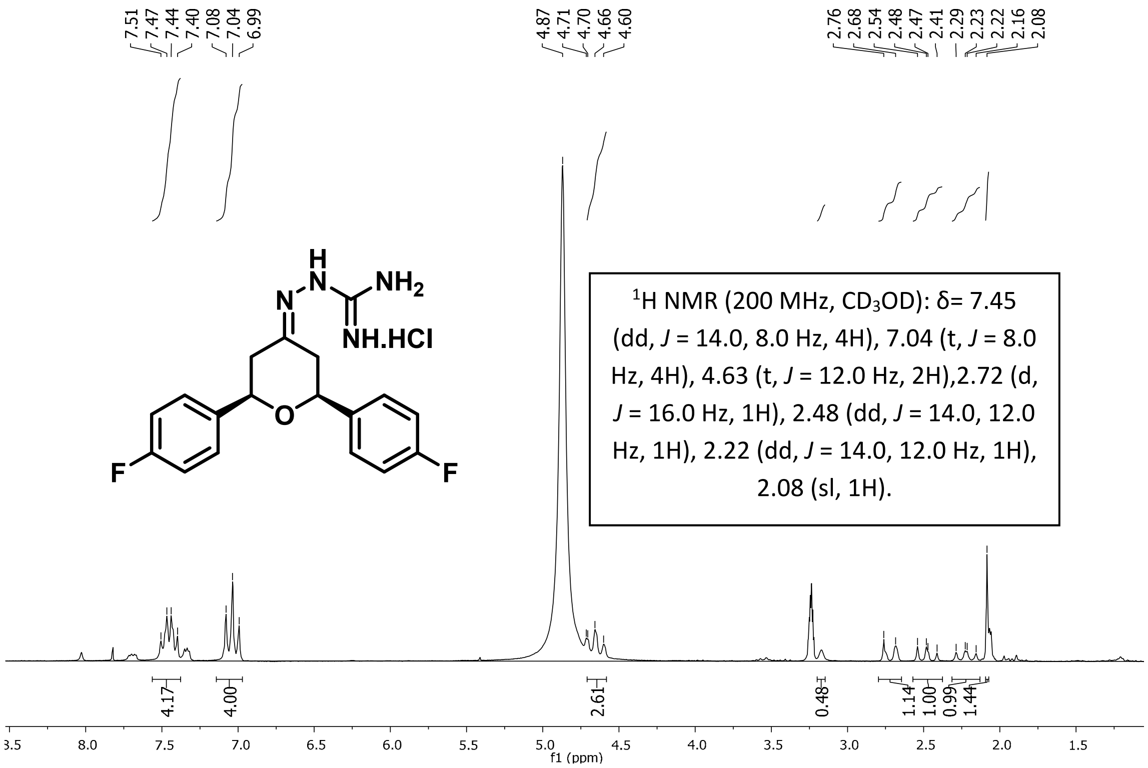

Figure S34. ^{1}H NMR (CD_3OD, 200 MHz) of 2-((cis-2,6-bis(4-fluorophenyl)dihydro-2H-pyran-4(3H)-ylidene)hydrazinecarboximidamide hydrochloride (**3**).

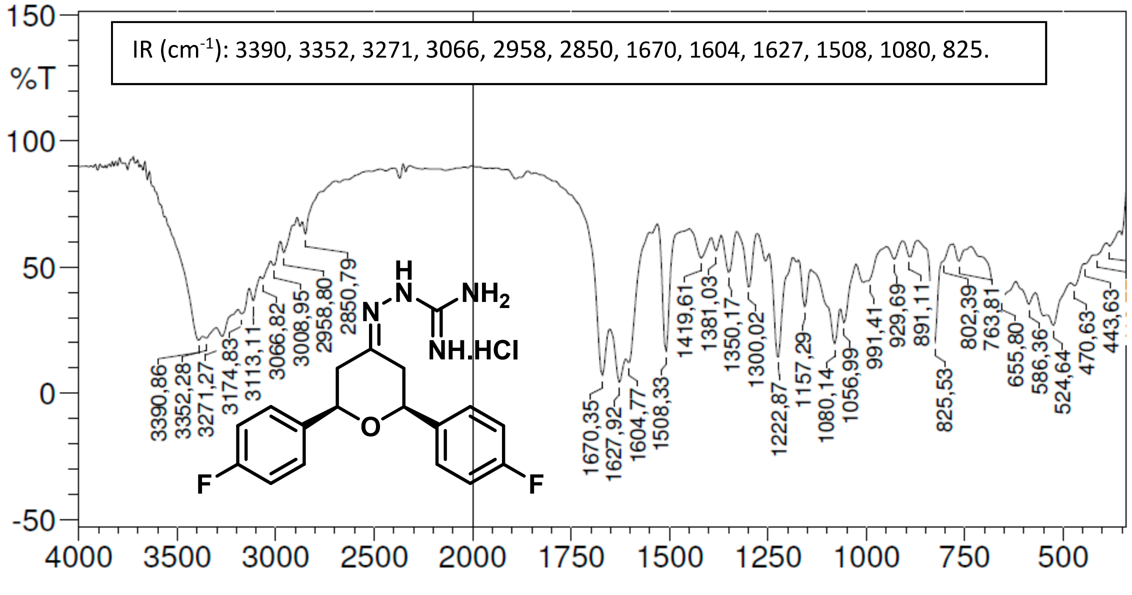

Figure S35. IR (KBr, cm^{-1}) of 2-((cis-2,6-bis(4-fluorophenyl)dihydro-2H-pyran-4(3H)-ylidene)hydrazinecarboximidamide hydrochloride (**3**).

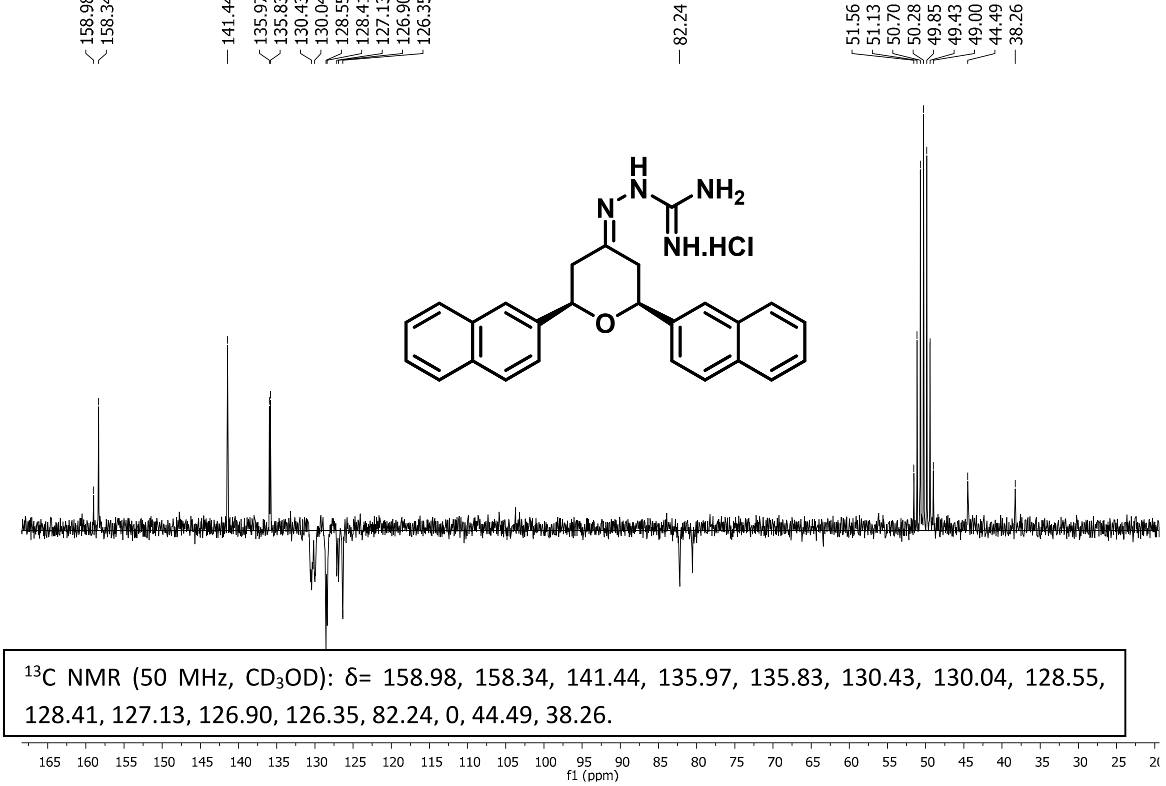

Figure S36. ^{13}C NMR-APT (CD_3OD, 50 MHz) of 2-((cis,trans)-2,6-di(naphthalen-2-yl)dihydro-2H-pyran-4(3H)-ylidene)hydrazinecarboximidamide hydrochloride (**4**).

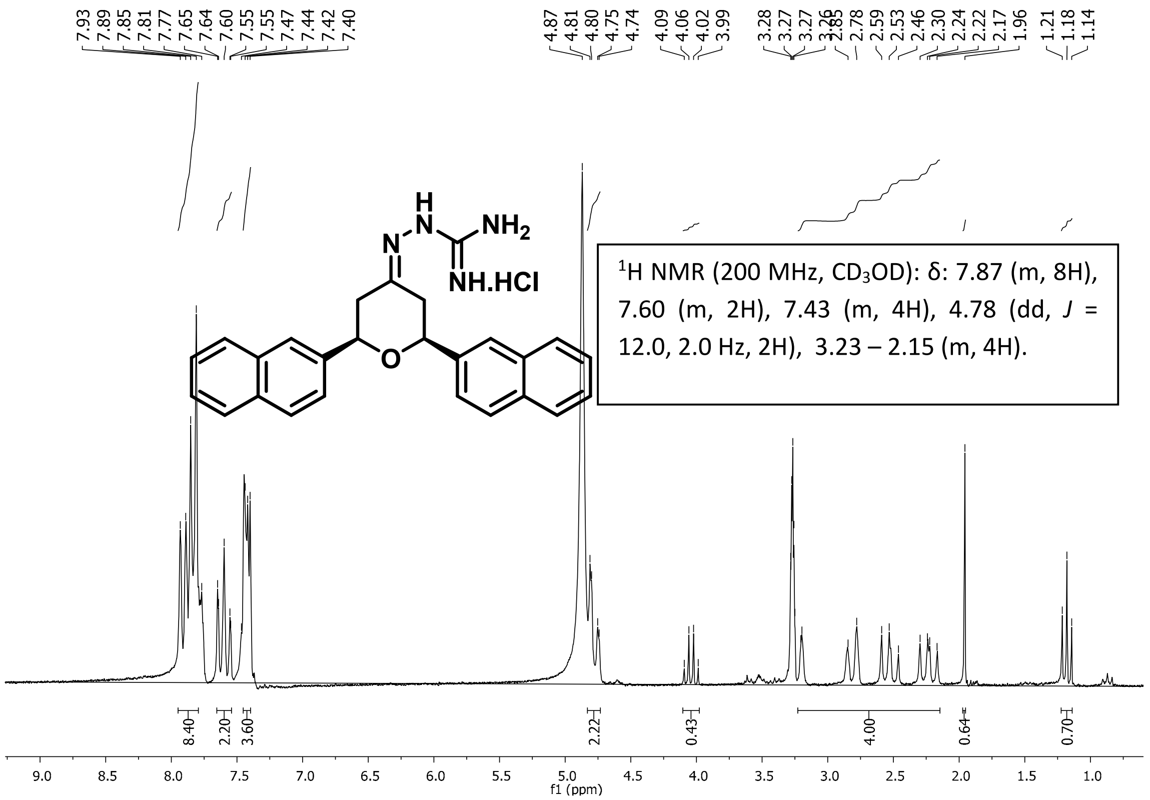

Figure S37. ^{1}H NMR (CD_3OD, 200 MHz) of 2-((cis,trans)-2,6-di(naphthalen-2-yl)dihydro-2H-pyran-4(3H)-ylidene)hydrazinecarboximidamide hydrochloride (**4**).

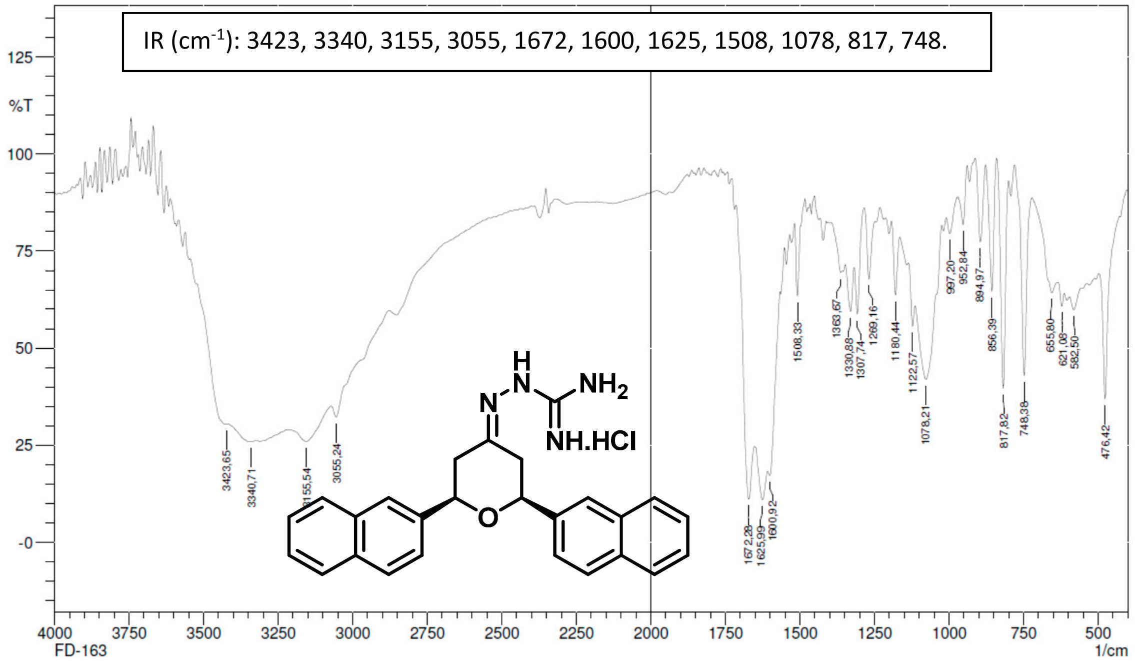

Figure S38. IR (KBr, cm^{-1}) of 2-((cis,trans)-2,6-di(naphthalen-2-yl)dihydro-2H-pyran-4(3H)-ylidene)hydrazinecarboximidamide hydrochloride (**4**).

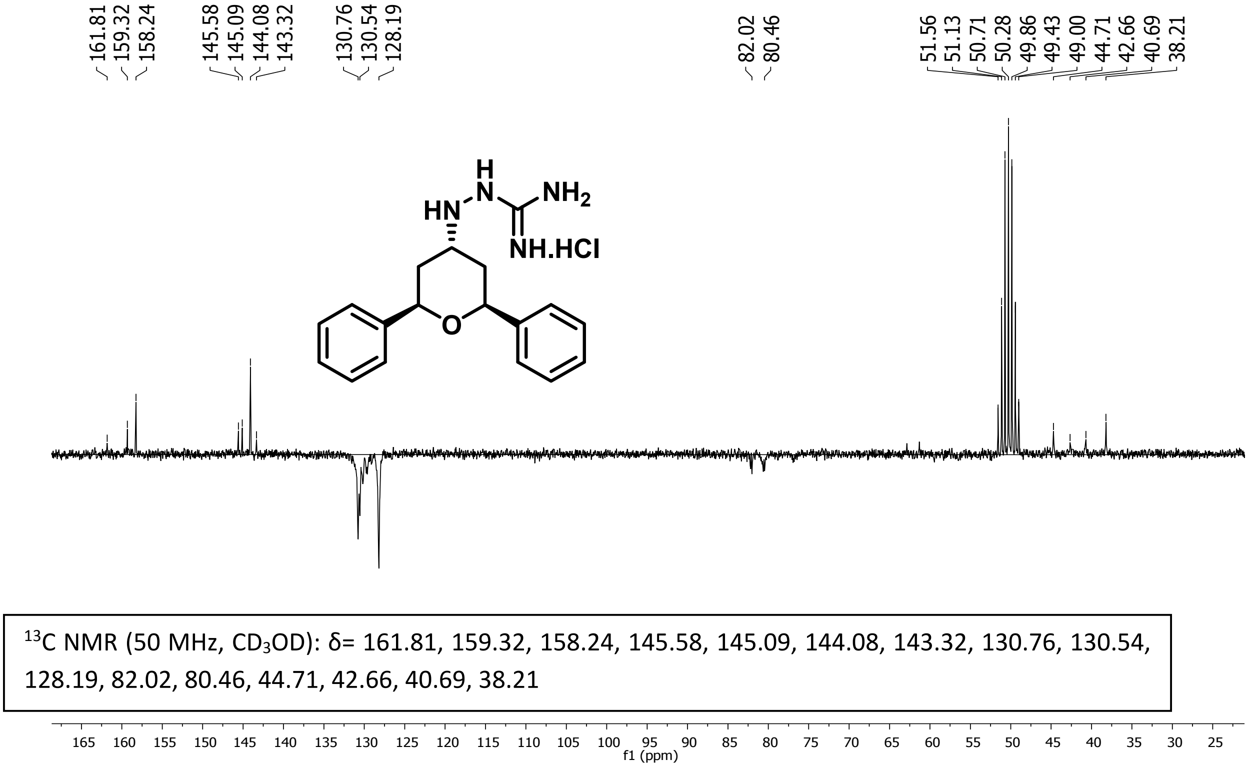

Figure S39. ^{13}C NMR-APT (CD_3OD, 50 MHz) of 2-((cis,trans)-2,6-diphenyltetrahydro-2H-pyran-4-yl)hydrazinecarboximidamide hydrochloride (**5**).

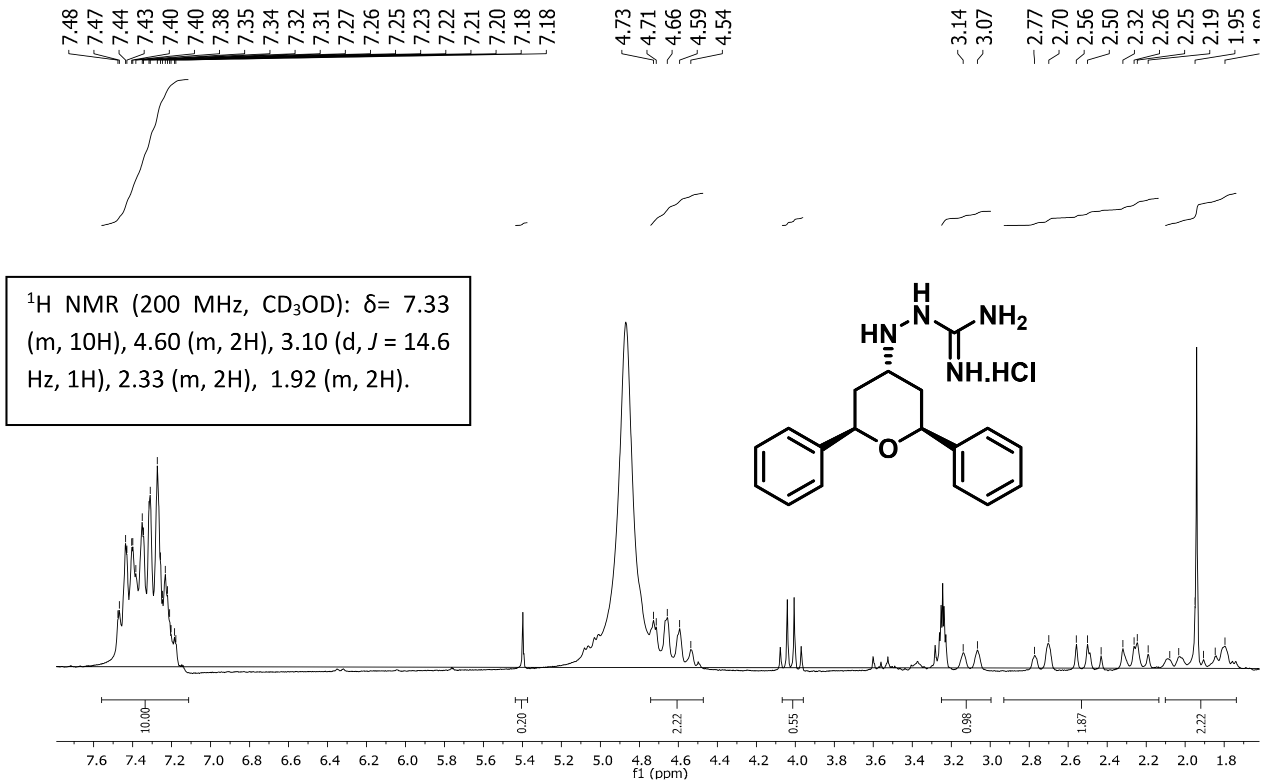

Figure S40. ^{1}H NMR (CD$_3$OD, 200 MHz) of 2-((cis,trans)-2,6-diphenyltetrahydro-2H-pyran-4-yl)hydrazinecarboximidamide hydrochloride (**5**).

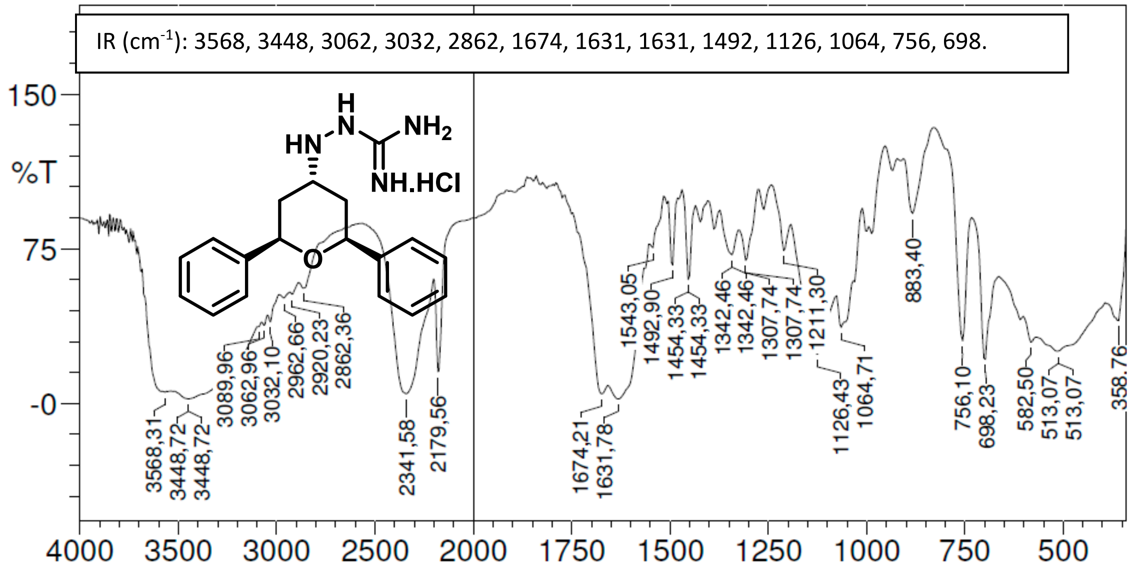

Figure S41. IR (KBr, cm^{-1}) of 2-((cis,trans)-2,6-diphenyltetrahydro-2H-pyran-4-yl)hydrazinecarboximidamide hydrochloride (**5**).

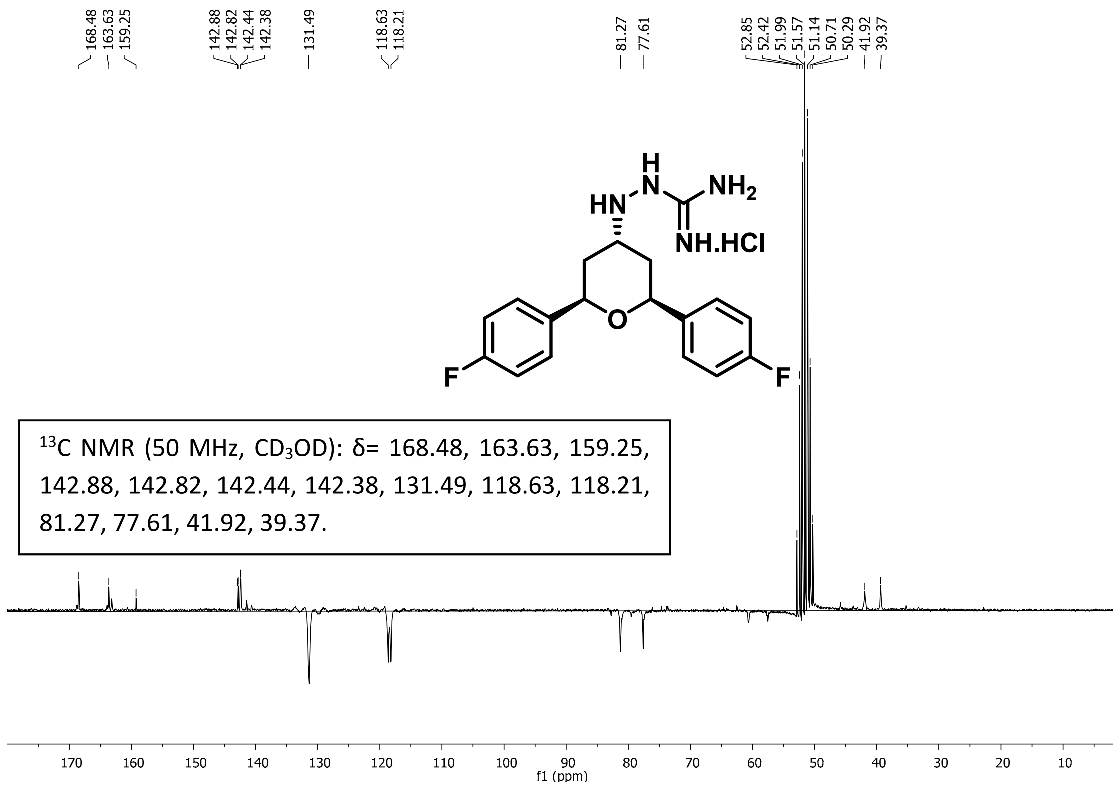

Figure S42. ^{13}C NMR-APT (CD_3OD, 50 MHz) of 2-((cis,trans)-2,6-bis(4-fluorophenyl)tetrahydro-2H-pyran-4-yl)hydrazinecarboximidamide hydrochloride (**6**).

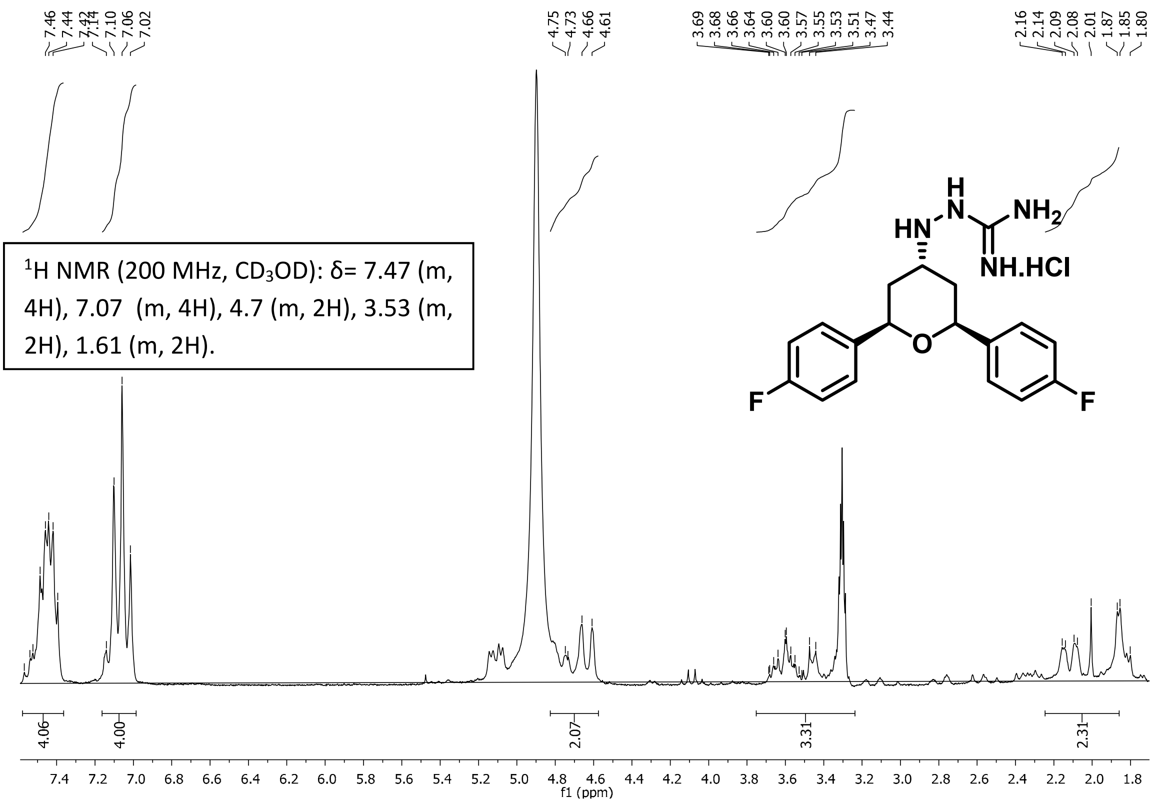

Figure S43. ^{1}H NMR (CD_3OD, 200 MHz) of 2-((cis,trans)-2,6-bis(4-fluorophenyl)tetrahydro-2H-pyran-4-yl)hydrazinecarboximidamide hydrochloride (**6**).

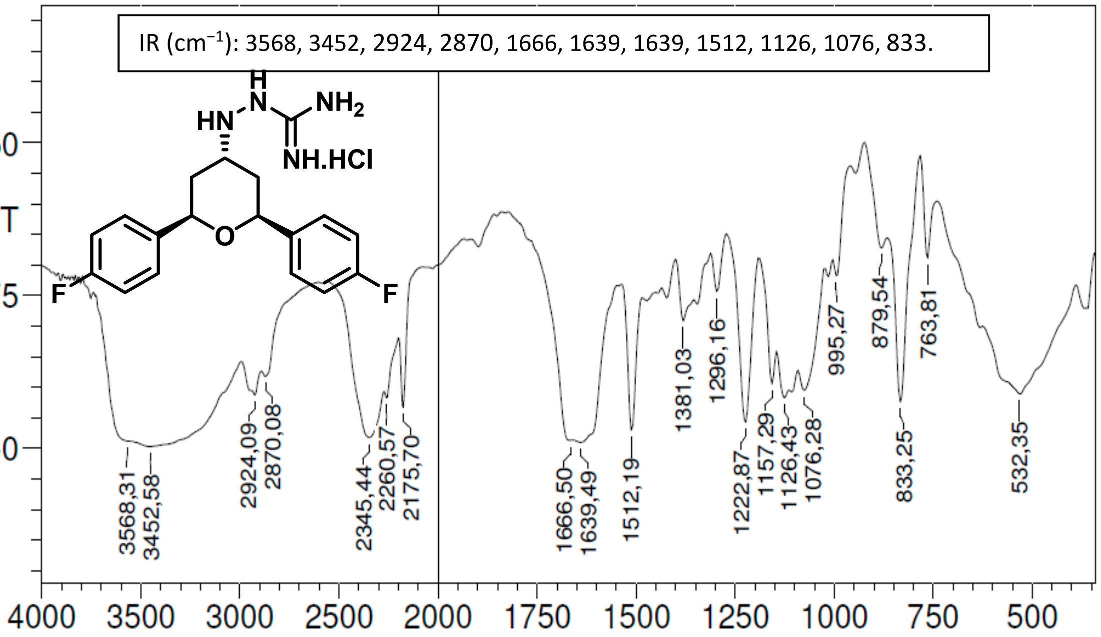

Figure S44. IR (KBr, cm^{-1}) of 2-((cis,trans)-2,6-bis(4-fluorophenyl)tetrahydro-2H-pyran-4-yl)hydrazinecarboximidamide hydrochloride (**6**).

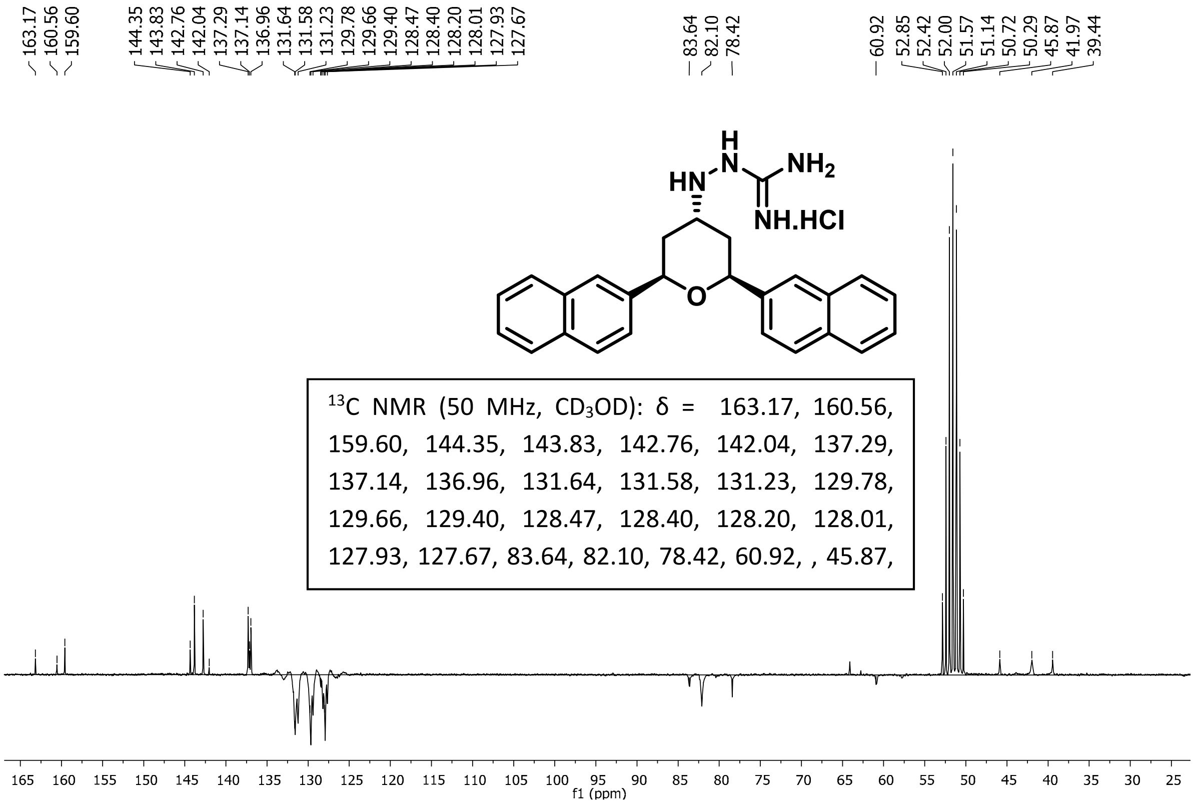

Figure S45. ^{13}C NMR-APT (CD_3OD, 50 MHz) of 2-((cis,trans)-2,6-di(naphthalen-2-yl)tetrahydro-2H-pyran-4-yl)hydrazinecarboximidamide hydrochloride (**7**).

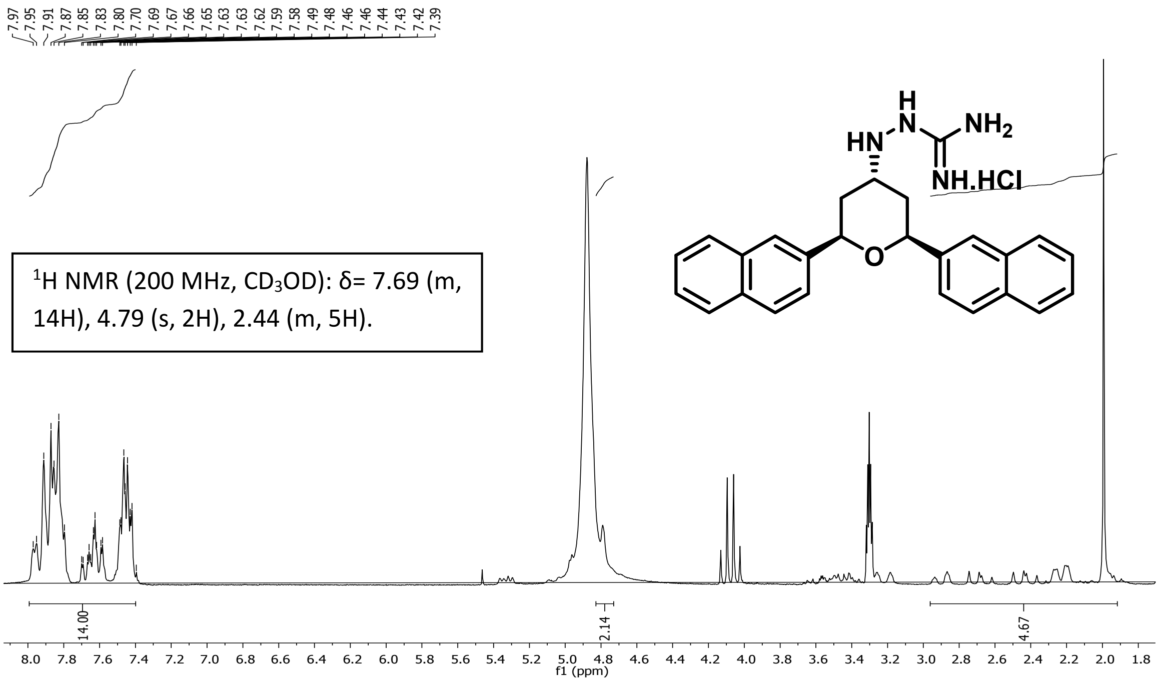

Figure S46. ^{1}H NMR (CD_3OD, 200 MHz) of 2-((cis,trans)-2,6-di(naphthalen-2-yl)tetrahydro-2H-pyran-4-yl)hydrazinecarboximidamide hydrochloride (**7**).

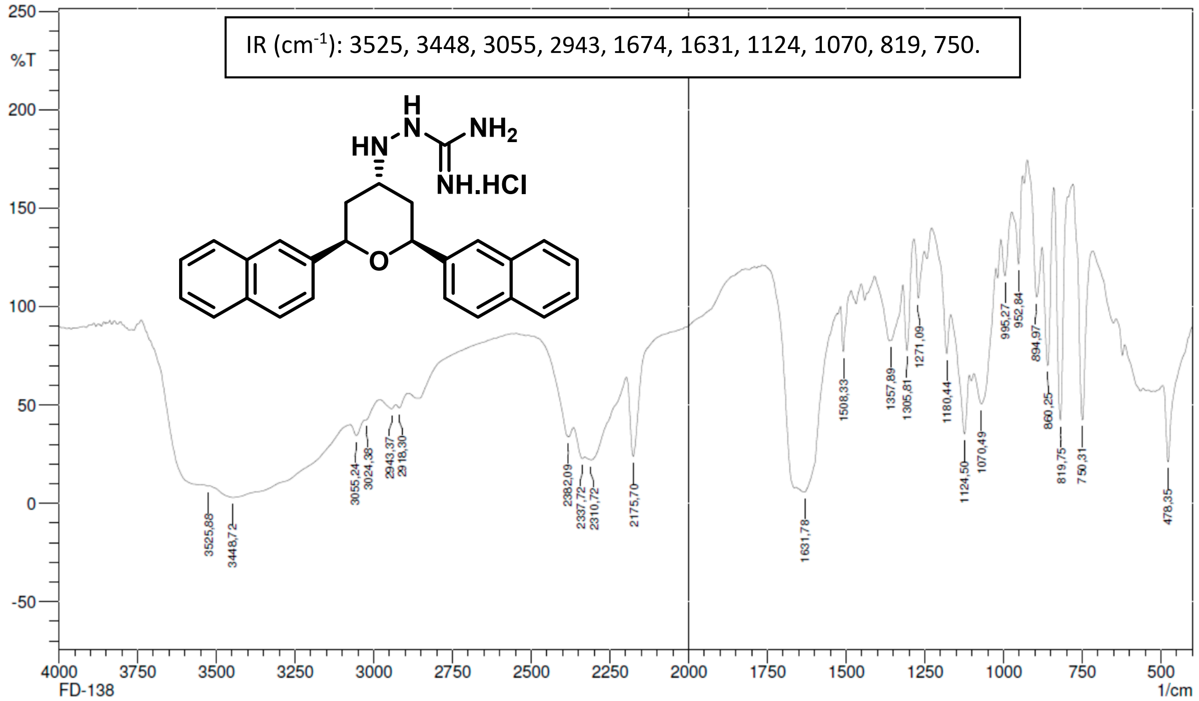

Figure S47. IR (KBr, cm^{-1}) of 2-((cis,trans)-2,6-di(naphthalen-2-yl)tetrahydro-2H-pyran-4-yl)hydrazinecarboximidamide hydrochloride (**7**).

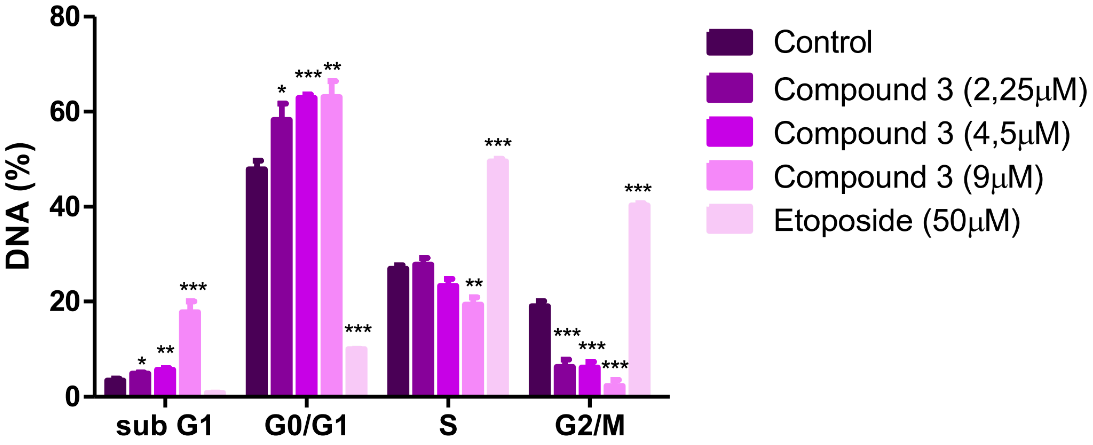

Figure S48. Effects of compound 3 in K562 cell cycle distribution, after 24 h. Cell cycle analysis was quantified by Flow cytometer on FL2-A, 10,000 events were recorded in each experiment. Data are expressed as percentage values. Each result was performed in duplicate, and data are reported as mean ± SD of at least three independent experiments. *P < 0.05; **<0.01; ***P < 0.001 *vs* control group.

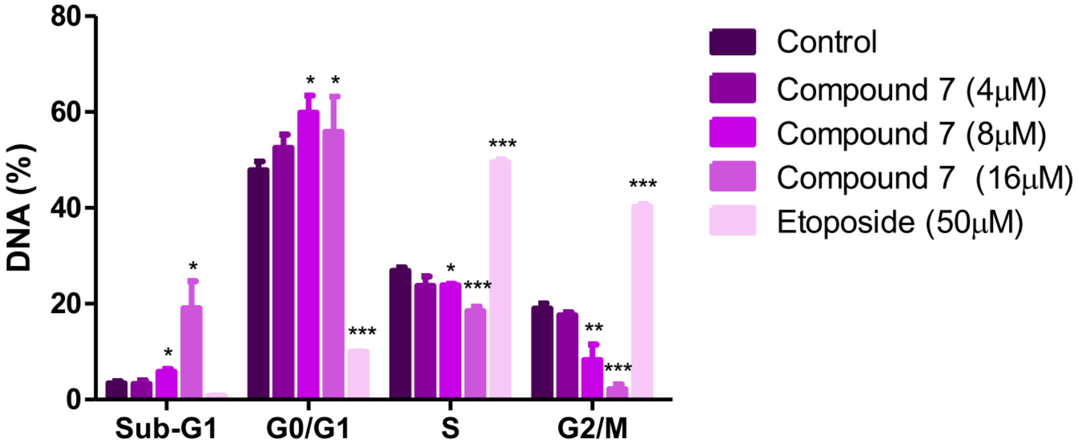

Figure S49. Effects of compound 7 in K562 cell cycle distribution, after 24 hours. Cell cycle analysis was quantified by Flow cytometer on FL2-A, 10000 events were recorded in each experiment. Data are expressed as percentage values. Each result was performed in duplicate, and data are reported as mean ± SD of at least three independent experiments. *P < 0.05; **<0.01; ***P < 0.001 vs control group.
